# Highly efficient CRISPR-mediated large DNA docking and multiplexed prime editing using a single baculovirus

**DOI:** 10.1093/nar/gkac587

**Published:** 2022-07-08

**Authors:** Francesco Aulicino, Martin Pelosse, Christine Toelzer, Julien Capin, Erwin Ilegems, Parisa Meysami, Ruth Rollarson, Per-Olof Berggren, Mark Simon Dillingham, Christiane Schaffitzel, Moin A Saleem, Gavin I Welsh, Imre Berger

**Affiliations:** BrisSynBio Bristol Synthetic Biology Centre, Biomedical Sciences, School of Biochemistry, 1 Tankard's Close, University of Bristol, Bristol BS8 1TD, UK; BrisSynBio Bristol Synthetic Biology Centre, Biomedical Sciences, School of Biochemistry, 1 Tankard's Close, University of Bristol, Bristol BS8 1TD, UK; BrisSynBio Bristol Synthetic Biology Centre, Biomedical Sciences, School of Biochemistry, 1 Tankard's Close, University of Bristol, Bristol BS8 1TD, UK; BrisSynBio Bristol Synthetic Biology Centre, Biomedical Sciences, School of Biochemistry, 1 Tankard's Close, University of Bristol, Bristol BS8 1TD, UK; The Rolf Luft Research Center for Diabetes and Endocrinology, Karolinska Institutet, SE-171 76 Stockholm, Sweden; BrisSynBio Bristol Synthetic Biology Centre, Biomedical Sciences, School of Biochemistry, 1 Tankard's Close, University of Bristol, Bristol BS8 1TD, UK; Bristol Renal, Bristol Medical School, Dorothy Hodgkin Building, Whitson street, Bristol BS1 3NY, UK; The Rolf Luft Research Center for Diabetes and Endocrinology, Karolinska Institutet, SE-171 76 Stockholm, Sweden; BrisSynBio Bristol Synthetic Biology Centre, Biomedical Sciences, School of Biochemistry, 1 Tankard's Close, University of Bristol, Bristol BS8 1TD, UK; BrisSynBio Bristol Synthetic Biology Centre, Biomedical Sciences, School of Biochemistry, 1 Tankard's Close, University of Bristol, Bristol BS8 1TD, UK; Bristol Renal, Bristol Medical School, Dorothy Hodgkin Building, Whitson street, Bristol BS1 3NY, UK; Bristol Renal, Bristol Medical School, Dorothy Hodgkin Building, Whitson street, Bristol BS1 3NY, UK; BrisSynBio Bristol Synthetic Biology Centre, Biomedical Sciences, School of Biochemistry, 1 Tankard's Close, University of Bristol, Bristol BS8 1TD, UK; Max Planck Bristol Centre for Minimal Biology, School of Chemistry, University of Bristol, Cantock's Close, Bristol BS8 1TS, UK

## Abstract

CRISPR-based precise gene-editing requires simultaneous delivery of multiple components into living cells, rapidly exceeding the cargo capacity of traditional viral vector systems. This challenge represents a major roadblock to genome engineering applications. Here we exploit the unmatched heterologous DNA cargo capacity of baculovirus to resolve this bottleneck in human cells. By encoding Cas9, sgRNA and Donor DNAs on a single, rapidly assembled baculoviral vector, we achieve with up to 30% efficacy whole-exon replacement in the intronic β-actin (*ACTB*) locus, including site-specific docking of very large DNA payloads. We use our approach to rescue wild-type podocin expression in steroid-resistant nephrotic syndrome (SRNS) patient derived podocytes. We demonstrate single baculovirus vectored delivery of single and multiplexed prime-editing toolkits, achieving up to 100% cleavage-free DNA search-and-replace interventions without detectable indels. Taken together, we provide a versatile delivery platform for single base to multi-gene level genome interventions, addressing the currently unmet need for a powerful delivery system accommodating current and future CRISPR technologies without the burden of limited cargo capacity.

## INTRODUCTION

CRISPR/Cas represents a game-changing, Nobel prize winning gene editing tool (1). A programmable DNA nuclease (Cas9) is precisely guided to a specific DNA locus by means of a short single guide RNA (sgRNA) to elicit double strand DNA breaks (DSBs) subsequently repaired through non-homologous end-joining (NHEJ) introducing small insertions-deletions (indels), giving rise to frameshift mutations and functional gene knock-outs (KOs) (2). Unpredictable indels that likewise occur are however undesirable in the context of therapeutic gene editing. Precise gene editing in contrast is typically achieved through homology directed repair (HDR) by providing a DNA template flanked by homology arms of variable length (2,3), resulting in precise gene correction or knock-in (KIs) (2). HDR activity however is intrinsically low and mostly restricted to S/G2 cell cycle phases (4–6), reducing the efficiency of the desired gene editing outcome. Significant effort is being made to increase the efficacy of CRISPR-HDR by means of small-molecule NHEJ inhibitors (7), cell cycle stabilised Cas9 variants (8,9) and other strategies (10,11), however, gene editing efficiency *in vivo* has remained low.

More recently, homology-independent targeted integration (HITI), was shown to efficiently induce base pair precise KIs in both dividing and non-dividing cells (12–14 by exploiting NHEJ and Cas9 cleaved donors, with exciting potential for gene editing applications *in vivo* (12,15). Moreover, single base substitutions can also be achieved by using base editors (BEs) involving catalytically impaired Cas9 variants fused to cytosine or adenine deaminase (16,17) and, more recently, prime editors (PEs) using Cas9 nickase fused to reverse transcriptase, achieving genomic interventions with little to no indels and reducing the risks associated with DSBs (18). BE, PE and HITI share a reliance on multiple functional DNA and protein elements that must be simultaneously delivered into target cells. This limits their applicability, particularly for future *bona fide* therapeutic interventions that necessitate a systemic approach and where co-transfection of plasmids and proteins, and likewise co-infection of viral vectors, will be problematic or unfeasible. In summary, the large DNA cargo capacity required for implementing these next-generation genomic interventions *in vivo* stands at odds with the limited cargo capacity of available technology, including the currently dominating adeno-associated virus (AAVs) and lentivirus (LVs) vector systems (19). In response to the CRISPR delivery challenge, new viral delivery vectors, with higher DNA cargo capacity, transduction efficiency and safety features are urgently required (20). To address this unmet need, we developed an *ad hoc* method for rapid generation of customizable baculoviral-vectors (BVs) for precise genome-engineering applications (MultiMate). BVs have a heterologous DNA cargo capacity far exceeding AAV and LV (19,21,22) and are widely used to transduce mammalian cells and living organisms (21–24. However, early attempts at CRISPR delivery using BVs have not provided significant benefits over other delivery systems, so far resulting in only modest gene editing efficiencies (21,25).

Here, we deploy a single baculoviral vector (BV) encoding all the required components, achieving high efficiency HITI, single and multiplexed prime editing in a range of human cell lines. We exploit our approach to correct a genetic defect in SRNS patient derived podocytes. By achieving site-specific integration of very large DNA payloads and multiplexed prime editing mediated trinucleotide insertion at four different loci we unlock baculovirus as a vector of choice for next-generation genome engineering approaches.

## MATERIALS AND METHODS

### Gibson assembly of DNA elements

An extensive list of constructs sequences and assembly strategies is provided in Supplementary Table S1. ENTR and DEST vectors were generated using Gibson assembly (NEB Builder Hi-Fi DNA assembly #E2621S) following manufacturer's instructions. Fragments were mixed with a backbone to insert ratio of 1:1 (>3 fragments), 1:2 (<2 fragments), 1:5 (fragments < 300 bp) in 5 μl total volume and supplemented with 5 ul 2xNEB Builder Hi-Fi mix followed by incubation at 50°C for 1 h. 2 ul of the assembly mix were transformed into homemade electrocompetent Top10 or Pir^+^*Escherichia coli*. followed by recovery at 37°C while shaking for 1 h and plating on LB/agar plates with the appropriate antibiotics. All the precursors vectors generated in this study were assembled using Gibson assembly of synthetic DNA fragments, oligonucleotides, digested vectors or PCRs amplified with Herculase II fusion (Agilent# 600675). A portion of pENTR-D-TOPO (Invitrogen) was used as backbone for all the pMMK ENTR vectors. attL/R sites on pMMK ENTR vectors were obtained by overlap extent PCRs of long oligonucelotides. attR1-Ccdb-Chlo-attR2 cassettes for generating pMMACE DEST were PCR amplified from pInducer-20 (26). pMMDS DEST was generated by replacing the Chloramphenicol cassette with Ori^ColE1^ PCR amplified from pACEMam1 (21). H2B-iRFP was PCR amplified from pCAG-H2BtdiRFP-IP (27), EYFP-Tubulin and mTFP1-Actin were amplified from 5-colours MultiBac vectors (21). pMDK 7 kb and 12 kb were generated by cloning promoterless and ATG-less portions of SMG1(NM_015092.5) CDS. Mito-mCherry was generated by fusing mCherry from 7TGC (28) to the COX8 mitochondrial targeting sequence from 5-colours MultiBac vectors (21). CyOFP1 (29) fused to the endoplasmic reticulum targeting sequence (ER) was synthesised by Twist Bioscience. mAG β-catenin was obtained through Gibson assembly of PCR amplified mAG from pL-EF1a mAG-hGeminin (30), and β-catenin from pL-EF1a β-catenin SV40 Puro (31). Golgi targeting sequence (GTS) mTaBFP was synthesised by Twist Bioscience. polH or p10 cassettes were amplified from MultiBac vectors (32,33), CCT isoforms were PCR amplified from synthetic vectors from GenScript. SpCas9 was PCR amplified from px459 (2), sgRNAs cloning was performed by overlap extent PCRs of hU6 and scaffold fragments amplified from px459 (2). HDR and HITI-2c templates were amplified by Gibson assembly of genomic DNA fragments, mCherry was amplified from 7TGC (28), T2A Puro from px459 (2). sgRNAs target in the HITI-2c donors were included as overlapping ends between fragments. polH/J23119 AcrII4 (34) was synthesised by Twist Bioscience. In the HITI-2c payloads for safe-harbour integration, mCherry T2A Puro was replaced with T2A mCherry P2A Puro. P2A was generated by overlap extent PCR of long oligonucleotides. The loxP site was PCR amplified from pACEBac1 (32,33), Hygromycin, IRES and eGFP were PCR-amplified from p1494 vectors (35). Increasing cargo sizes were iteratively added by CRE-mediated recombination of pMDK 7kb and pMDK 12 kb (described above). CMV wild-type NPHS2 was amplified from pLenti CMV WT Podocin myc Blast (courtesy of Gavin Welsh). PE2 was obtained by restriction digestion from pCMV PE2 (18) and the HEK3 PegRNA was PCR amplified from pU6-Sp-pegRNA-HEK3_CTT_ins (18). HEK3 nicking sgRNA (18) was PCR amplified from a synthetic DNA fragment obtained from Twist Bioscience. aeBlue (36) was synthesised by Twist Bioscience and VSVG-G was amplified from pMD2.G (Didier Trono lab, Addgene plasmid # 12259). DNMT1, EMX1, RNF2 and RUNX1 PegRNAs and nicking sgRNAs were amplified from synthetic fragments obtained from Twist Bioscience and assembled by Gibson cloning in their respective vectors.

### LR recombination

LR recombination was carried out using LR Clonase II (Thermo Fisher #11791020) or LR Clonase II plus (Thermo Fisher #12538120). Although LR Clonase II plus is specifically designed for MultiSite Gateway recombination, LR Clonase II worked with comparable efficiency in our hands. LR reactions were carried out following manufacturer's instructions. One DEST and four ENTR vectors were diluted to 20 femtomoles/μl each in TE buffer pH 8.0 (Thermo Fisher #12090015). 1 ul of each diluted vector was added to a 0.2 ml PCR tube with 2 ul LR Clonase II and 3 ul TE buffer, followed by a brief spin and incubation at 25°C for 16 hours. The next day the reaction was terminated by addition of 1 ul proteinase K (provided with LR Clonase II enzymes) and incubation at 37°C for 10 min. 2–3 ul were transformed into homemade electrocompetent Top10 or Pir^+^*E. coli*, followed by 2 h recovery at 37°C and plating on LB/agar plates with the appropriate antibiotics. LR recombination products were predicted using APE (37) with custom recombination reactions. To quickly load MultiMate LR reaction prototype in APE, the following code can be copied and used in Tools/Recombination Reaction Editon/New reaction from clipboard:

MultiMate LR Reaction for APE:

{ApE recombination reaction:}{MultiMate LR Reaction (1-3-4-5-2)} {{{pMMK ENTR 1} {pMMK ENTR 2} {pMMK ENTR 3} {pMMK ENTR 4} DEST} {attB1 0 attB3 0 attB4 0 attB5 0 attB2 1}}

When imported as GenBank or Fasta files, the MultiMate LR reaction in APE will automatically recognize pMMK ENTR1-4 vectors and one DEST donors when launched through Tools/Recombination tools. For manual prediction of MultiMate assembly products, a list of the attL/R sequences and their attB products is provided in Supplementary Table S3.

### Plasmid propagation and stability

Upon LR recombination, plasmids assembled through MultiMate were propagated in Top10 or DH5α *E. coli* under standard culturing conditions (37°C) in LB broth supplemented with the appropriate antibiotics. We assembled plasmids up to 33 kb in size and containing a number of identically repeated elements (e.g. promoters, terminators), but did not observe spontaneous recombination with any of our assemblies.

Recombination in *E. coli* systematically occurred for MultiMate HITI-2c vectors in absence of an AcrII4 module. Additionally, empty pMMK_ENTR4 and pMKK_ENTR_AcrII4 modules occasionally recombined during bacterial propagation. This is due to the similarity and proximity of attL5/2 in pMKK_ENTR4 and to the constitutive bacterial expression of AcrII4 in pMMK_ENTR_AcrII4. When the distance between attL5/L2 was increased by cloning an intervening cassette (e.g. pMMK4_CMV_eGFP), no recombination events were observed for pMMK_ENTR_4 modules. When the pMMK_ENTR_AcrII4 modules were assembled into MultiMate HITI-2c vectors, no additional recombination of the AcrII4 cassette was observed.

### Cre-mediated recombination of DNA elements

One acceptor and one or multiple donor vectors were assembled using Cre-mediated recombination as previously described (33,38–39). One acceptor and one or more donors were mixed with a ratio of 1:1.1 in distilled H2O with 0.5 ul (7.5 U) of CRE recombinase (NEB # M0298M) and 1 ul Cre buffer (provided with CRE recombinase) to a final volume of 10 ul in distilled H_2_O. 500–1000 ng of total DNA were used for each reaction. Cre-reactions were incubated for 1 hour at 37°C, followed by heat inactivation at 70°C for 10 min. 2–3 ul were transformed into homemade electrocompetent Top10 *E. coli*, followed by 2 h recovery at 37°C and plating on LB/agar plates with the appropriate antibiotics. Cre-recombination products were predicted using Cre-ACEMBLER Vers. 2.0 (38).

### Cell culture methods

Human cells (HEK293T, HeLa, H4, RPE-1 hTERT and SH-SY5Y) were purchased from ATCC and propagated as adherent cultures in 60 or 100 cm dishes in a humidified incubator (37°C, 5% CO_2_). For passaging cells were washed with phosphate saline buffer (DPBS, Gibco # 14190144), detached using 0.25% Trypsin (Thermo Fisher #25200056) followed by a brief incubation at 37°C, centrifuged at 300× RCF and resuspended in fresh media in a new plate at the desired concentration. Podocyte cell culture was carried out as previously described (40). Suspension cultures of Sf21 insect cells were grown in 125 ml or 250 ml polycarbonate Erlenmeyer flasks with vent cap (CORNING, #431143, #431144) at 27°C in a shaking incubator. Sf21 were split every 2–3 days and maintained at concentrations between 0.5–2 × 10^6^ cells/ml. Origin and media formulation recipe for each cell line is reported in Supplementary Table S4.

For transfection in HEK293T, 2 × 10^5^ cells/well were seeded in multi-24 wells. Transfections were carried out using Polyfect (QIAGEN #301105), following manufacturer's instructions. Briefly 500 ng of DNA were resuspended in 25 ul of Optimem (Gibco #31985062), supplemented with 5 ul Polyfect and incubated for 15 min at room temperature. Transfection mix was resuspended with 100 ul of complete media and added dropwise to each well. Cells were cultured for at least 48 hours before assessing the phenotype (e.g. fluorescence markers expression).

For puromycin selection of HITI-2c edited cells, puromycin dihydrochloride (Gibco A1113803) was used at a final concentration of 1 μg/ml. Puromycin was added at 5–7 days post-transfection/transduction for 7 days. After selection, cells were maintained in absence of puromycin for at least 1 week prior downstream analysis. For puromycin/hygromycin selection of large DNA payload editing events, cells were first selected with puromycin as described above, followed by 10 days selection with 1 μg/ml puromycin and 250 μg/ml hygromycin (Hygromycin B, ThermoFisher #10687010). Upon completion of double selection, cells were maintained in absence of puromycin and hygromycin for at least 1 week prior downstream analysis.

### Baculovirus vector amplification

Assembled MultiMate vectors were shuttled on baculovirus genomes (bacmids) propagated in *E. coli* using Tn7 transposition. 200–1000 ng of MultiMate vector were transformed in chemically competent DH10-MultiBacMam-VSV-G (21), DH10-EMBacY (33) or commercial DH10Bac (ThermoFisher # 10361012) as previously described (33). DH10-EmbacY were used to generate baculoviruses for multiprotein expression in insect cells and low MOT transduction of HEK293T, DH10-MultiBacMam-VSV-G were used for high MOT human cells transduction.DH10Bac were used for high MOT transduction of single and multiplexed prime editing constructs in human cells, by providing an additional module encoding for VSV-G in insect cells. DH10Bac were included in this study as they are widely wide used,and to demonstrate their compatibility with MultiMate. DH10-MultiBacMam-VSV-G are however preferrable for mammalian cells transduction, as the VSV-G module is already integrated in the baculoviral genome (21), alleviating the need for additional DNA assembly. Bacteria were streaked on LB/Agar plates with Gentamycin, Kanamycin, Tetracyclin, IPTG and Bluo-Gal and incubated for 24-hours for blue-white screening. White colonies were picked and grown overnight in 3 ml of LB supplemented with Gentamycin/Kanamycin to extract bacmid DNA through alkaline lysis/ethanol precipitation as previously described (32,33).

For transfection in insect cells, 0.8–1 × 10^6^ Sf21 cells/well were seeded on multi-6 well plates in 3 ml of Sf-900 II media. 10 ul of purified bacmid were resuspended in 130 ul Sf-900 II media with 10 ul X-treme XP transfection reagent (Roche # 06366236001) and incubated at room temperature for 15 min. The entire transfection mix was added dropwise to a single well and cells were incubated at 27°C in a static incubator. V_0_ viral stocks were harvested collecting the supernatant of transfected cells 72–96 hours post transfection as previously described (19,32,47). 1–3 ml of V_0_ viral stocks was added to 10 ml of fresh Sf21 cells at 0.8 × 10^6^ cells/ml. Cells were cultured in 50 ml Falcon tubes while shaking at 27°C and counted every day to monitor cell proliferation and size using Luna cell-counter (LogosBio). Successfully infected cells displayed arrested proliferation and increased average cell size (13–14 μm control, 16–20 μm infected). V_1_ viral harvest were collected as previously described 2 days after proliferation arrest (DPA + 24) (19,32,47) by centrifugation at 4500 × rcf. 500 μl/1 ml of V_1_ viral stocks was added to 50 ml of fresh Sf21 cells at 0.8 × 10^6^ cells/ml in 125 ml Erlenmeyer flasks and cells were cultured at 27°C in a shaking incubator. V_2_ viral harvests were collected by centrifugation at 4500 × rcf, and concentrated 20 times by high-speed centrifugation at 11000 × rcf, followed by resuspension in DPBS supplemented with 3% heat inactivated FBS and 1% glycerol for storage at −80°C.

### Baculovirus vector titration and transduction

For BV expressing fluorescent markers in human cells, titration was performed as previously described (41). HEK293T were used to determine viral titers. Briefly 1 × 10^5^ cells/well were seeded in multi-48 wells plates in 200 ul of complete media. Concentrated virus was serially diluted in DPBS and 50 μl were dispensed to each well. Spinoculation (30′ at 600 × rcf at 27°C) was used to enhance transduction as previously reported (42). Twenty four hours after transduction, cells were analysed using flow-cytometry to determine the percentage of transduced cells. TU/ml values from dilutions giving <20% transduction efficiencies were averaged to estimate the titer as previously described (41) and using Supplementary Equation 1. For experiments in which different viral titers were used, multiplicity of transduction (MOT) was calculated as TU*/Cn*. Transduction in various cell lines was performed. 2 × 10^5^ cells per well were seeded in multi-48 wells chambers in 200 μl DPBS, 50 ul of diluted virus at the appropriate multiplicity of transduction (MOT) were added and cells were spinoculated as described. After spinoculation, viral supernatant was removed, the cells were detached by trypsinization and replated to multi-24 wells. For podocytes transduction, BacMam enhancer (Thermo Fisher # B10107) was added at 1:1000 dilution for 24 h where indicated and removed by 3× DPBS washes.

### Confocal and widefield imaging

Confocal images were acquired using a Leica Sp8 equipped with 405, 458, 476, 488, 496, 514, 561, 594, 633 nm laser lines and 37°C stage. For time lapse confocal experiments on living cells the stage was supplemented with 5% CO_2_. For higher magnification, cells were plated on Lab-Tek borosilicate multi-8 wells (Thermo Fisher # 155411). For experiments in which transduced cells expressed more than three different fluorochromes, laser intensity and detection filters were adjusted to reduce spectral overlap using individual fluorescence controls transfections. Widefield and phase contrast images were acquired using a Leica DMI6000 equipped with excitation/emission filters optimised for DAPI, GFP, Rhodamine, Texas Red and Far red.

### Flow cytometry analysis

For flow-cytometry analysis cells were trypsinised and resuspended in complete media supplemented with 3 μM DRAQ7 (Abcam #ab109202) to counterstain dead cells. Cells were analysed on a Becton Dickinson LSR Fortessa X-20 (4 lasers 16 colours, HTS), fluorochromes were detected as follow: eGFP and EYFP (FITC-A), mCherry (PECF594-A), mTagBFP (BV421-A), DRAQ7 (AlexaFluor700-A). SSC-A and FSC-A were used to discriminate single cells and cell populations by size. FlowJo X was used to analyse FCS files. All data represented are percentages of live single cells (DRAQ7-).

### PCR genotyping, Sanger sequencing and deconvolution

Genomic DNA was extracted with QIAamp DNA Mini Kit (QIAGEN # 51306) following manufacturer's instruction. A list of the predicted gene editing outcome sequences and genotyping oligos is provided in Supplementary Table S2. PCRs were performed using KAPA2G Fast Genotyping mix (SigmaAldrich # KK5621) following manufacturer's instruction. Amplicons were run on 0.8% agarose gels, purified using QIAquick Gel Extraction Kit (QIAGEN # 28706) and eluted in distilled ddH_2_O. For Sanger sequencing 15 ul of purified PCR at 5–10 ng/μl were mixed with 2 μl of diluted (10 μM) sequencing primer and sent to an external sequencing service (Eurofins). Electropherograms (.ab1) from parental and transduced cells were fed into ICE (43) from Synthego for sequence deconvolution and indels/knock-in estimation.

### Western blot

Total protein extracts from HEK293T were obtained by lysing the cells with ice-cold RIPA Buffer (Thermo Fisher # 89901) supplemented with protease inhibitors (Thermo Fisher # 78429) for 30′ on ice. Insoluble material was pelleted by centrifugation at 16 000 × rcf at 4°C for 5 min. Total protein extracts from insect cells were obtained as previously described (32). Proteins concentrations were determined using Nanodrop. 10 μg protein/sample were stained with Laemmli buffer, boiled at 95°C for 5 min, separated using pre-cast NuPage 4–12% Bis–Tris SDS-Gels (Thermo Fisher # NP0321BOX) and transferred to PVDF membranes using iBlot. Membranes were blocked with 5% non-fat dry milk in T-TBS (50 mM Tris–Cl, pH 7.6, 150 mM NaCl, 0.5% Tween) for 1 h at room temperature. Membranes were incubated with primary antibodies diluted 1:1000 in T-TBS 5% milk overnight at 4°C while rocking, followed by two T-TBS washes and incubation with HRP-conjugated secondary antibody diluted 1:2000 in T-TBS 5% milk for 1 h at room temperature. Membranes were washed again with T-TBS and developed using Pierce ECL reagents (Thermo Fisher # 34579) following manufacturer's instructions. Finally, membranes were imaged using MyECL Imager. A list of the primary and secondary antibodies used is provided in Supplementary Table S5.

### Immunofluorescence

For immunofluorescence, cells were seeded on glass coversleep and fixed with 4% paraformaldehyde (Figure 4G) or methanol (Figure 4H) for 15 min at room temperature or at −20°C, respectively. Cells were washed twice with DPBS following incubation in blocking solution containing 3% BSA (Sigma) and 0.1% Triton X-100 (Sigma) for 1 h at room temperature. The cells were then left overnight at 4°C in 0.5× blocking solution containing the primary antibody. The next day, the cells were washed three times with DPBS and then incubated with the secondary antibody for 1 h at room temperature in PBS. Coverslips were mounted on glass slides using Vectashield + DAPI (2bscientific # H-1200–10) and imaged using confocal microscopy. A list of the primary and secondary antibodies used is provided in Supplementary Table S5.

### CCT/TriC complex purification

Recombinant MultiMate-CCT BV was produced as previously described (33) and used to infect Sf21 insect cells at a cell density of 1.0 × 10^6^/ml in Sf-900 II medium. Cells were harvested 72–96 h after proliferation arrest by centrifugation at 1000 × g for 15 min. Cell pellets were resuspended in lysis buffer (50 mM HEPES–NaOH, 200 mM KCl, 10 mM Imidazole, 20% Glycerol, pH 7.5, supplemented with EDTA-free protease inhibitor (Sigma-Aldrich) and Benzonase (Sigma-Aldrich)) and lysed by short sonication. The lysate was cleared by centrifugation at 18 000 rpm, 4°C, in a F21-8 × 50y rotor (Thermo Fisher Scientific) for 60 min. The supernatant was loaded on a TALON column (Generon), equilibrated in TALON A buffer (50 mM HEPES–NaOH, 200 mM KCl, 10 mM Imidazole, 20% glycerol, pH 7.5) with a peristaltic pump. The column was washed with ten column volumes (CV) of TALON A buffer before eluting the bound protein complex with a step gradient of TALON B buffer (50 mM HEPES–NaOH, 200 mM KCl, 250 mM Imidazole, 20% glycerol, pH 7.5). The CCT protein complex was buffer exchanged in Heparin A buffer (50 mM HEPES–NaOH, 100 mM KCl, 10% glycerol, pH 7.5) while concentrating. It was then subjected to a Heparin column (GE Healthcare) and eluted with a 1 M KCl gradient in Heparin A buffer. Fully formed complexes and disassembled subunits were separated on a Superose 6 10/300 column (GE Healthcare) equilibrated in SEC buffer (20 mM HEPES–NaOH, 200 mM KCl, pH 7.5, 1 mM DTT, 10% glycerol). Peak fractions were pooled and concentrated and the purity of the CCT complex was analyzed by SDS-PAGE.

### Electron microscopy

For electron microscopy, copper grids with carbon coating (300 mesh, Electron Microscopy Sciences) were glow discharged for 10 s, and 5 μl of purified CCT was placed on the grids for 1 min. Afterwards the grid was washed for 15 s and floated onto a drop of filtered 3% uranyl acetate for 1 min. Excess solution on the grids was blotted off using filter paper between each step. Grids were visualized under a FEI Tecnai 20 transmission electron microscope (TEM), and digital micrographs were taken using a FEI Eagle 4K × 4K CCD camera. Particle picking and processing was performed using RELION 2 (44,45), 2D class averages were generated without applying symmetry or reference models.

## RESULTS

### Rapid assembly of highly modular baculovirus multigene vectors using MultiMate

**Figure 1.**
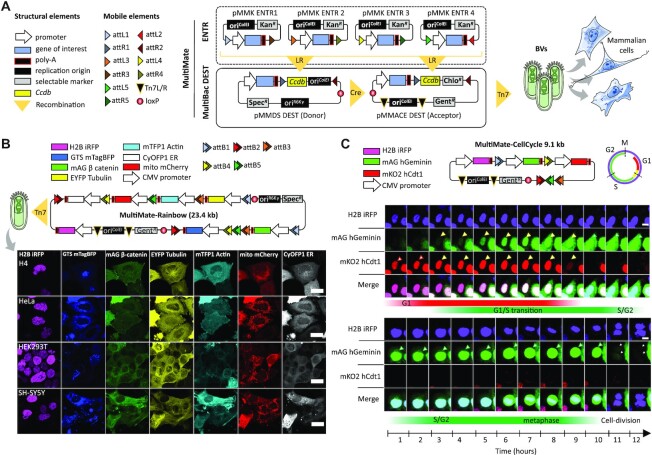
MultiMate enables rapid modular assembly of multifunctional DNA circuitry for efficient baculovirus-vectored delivery in human cells. (**A**) MultiMate assembly platform in a schematic view. Symbols are listed (left panel). attL/R flanked DNA from ENTR plasmid modules (upper box, dashed) are assembled on MultiBac-DEST (lower box) and further combined by *in vitro* Cre-mediated recombination to generate multicomponent MultiMate plasmids, maximally eliminating prokaryotic backbone DNA sequences. MultiMate plasmids are integrated in BVs customized for efficient delivery in human cells (right panel). (**B**) Confocal live cell imaging of H4, HeLa, HEK293T and SH-SY5Y 48 hours after transduction with MultiMate-Rainbow BV (upper panel) evidencing in all cells homogeneous sustained expression and correct subcellular localization of H2B-iRFP (nucleus), GTS-mTagBFP (Golgi), mAG-β-catenin (membrane and adherens junctions), EYFP-Tubulin (microtubules), mTFP1-Actin (cytoskeleton), mito-mCherry (mitochondria) and CyOFP1-ER (endoplasmic reticulum). Scalebar, 20 μm. (**C**) Twelve-hours confocal time-lapse imaging of live HeLa cells transduced with MultiMate-CellCycle BV (snapshots, Supplementary Video 1). Transduced cells constitutively express H2B-iRFP for DNA imaging. mAG-hGeminin and mKO2-hCdt1 are stabilized in S/G2 and G1 cell cycle stages, respectively. Upper panel shows G1/S transition, lower panel shows a dividing cell (arrows indicate tracked cells). Scalebar, 10 μm. DNA elements, plasmid topology and cell cycle schematics are illustrated (upper panel).

We have previously developed methods to rapidly assemble functional DNA elements into multicomponent circuitry in a baculoviral vector (BV) (39,46,47). Here, we optimized and fine-tuned our DNA assembly approach (MultiMate) by combining proven MultiSite Gateway technologies with MultiBac (32,33) recombination modalities to assemble with ease theoretically up to 25 distinct DNA elements of various sizes (Figure 1A, Supplementary Figure S1a–e, Supplementary Methods), importantly aiming to significantly reduce prokaryotic elements carried over into the baculovirus which can compromise vector integrity during manufacturing (48) (Supplementary Figure S1f). We validated the MultiMate system by expressing the eight subunit human chaperonin CCT/TRiC complex (49) in insect cells from a BV comprising MultiMate assembled DNA (23 kb) (Supplementary Figure S1g–j). We then assessed the ability of MultiMate to efficiently deliver large multicomponent DNAs for expression and live cells imaging of up to 7 fluorescently labelled proteins (MultiMate-Rainbow) in human cells (Figure 1B, Supplementary Figure S2a). MultiMate assembly yielded remarkably low error rates validating our approach (Supplementary Figure S2b). We deployed BVs harbouring 18 and 23.4 kb of MultiMate assembled functional DNA to efficiently transduce HEK293T, HeLa, H4 and SH-SY5Y leading to homogeneous expression and correct subcellular localization of all fluorescently labelled proteins (Figure 1B, Supplementary Figure S2c). Moreover, we created MultiMate-CellCycle (9.1 kb) as an improved cell cycle tracking tool implementing FUCCI reporters (50) as well as H2B-iRFP expression for accurate transduction efficiency monitoring and cell cycle stage assessment (Figure 1C, Supplementary Figure S2d, e). MultiMate-CellCycle BVs efficiently transduced HEK293T, HeLa, H4 and SH-SY5Y cells highlighting differences in their respective cell cycle progressions (Supplementary Figure S2f) and enabling 12-h time lapse imaging on living HeLa cells, allowing cell cycle tracking also during FUCCI-unstained stages (M-G1 transition) (Figure 1C, Supplementary Video 1). These results comprehensively validate the MultiMate assembly platform enabling a wide range of baculovirus-vectored applications.

### Baculovirus-vectored homology independent targeted integration (HITI)

To date, baculovirus-vectored gene editing approaches were confined to CRISPR-HDR of small insert DNAs with low efficacy (21,24–25). HITI toolkits using a viral vector required donor and Cas9/sgRNA to be split between two AAVs due to their limited cargo capacity (12), restricting successful gene editing to the fraction of co-infected cells. Moreover, manufacturing complete ‘all-in-one’ HITI vectors is not possible when viral packaging is performed in mammalian cells (typically HEK293T for AAV, LV), because simultaneous expression of Cas9 and sgRNA would inevitably excise the HITI donor, fatally compromising virus production. In marked contrast, BVs are manufactured in insect cells, where the mammalian promoters controlling Cas9 and sgRNA expression are poorly used and all-in-one HITI construct packaging into the BV is thus entirely feasible.

**Figure 2.**
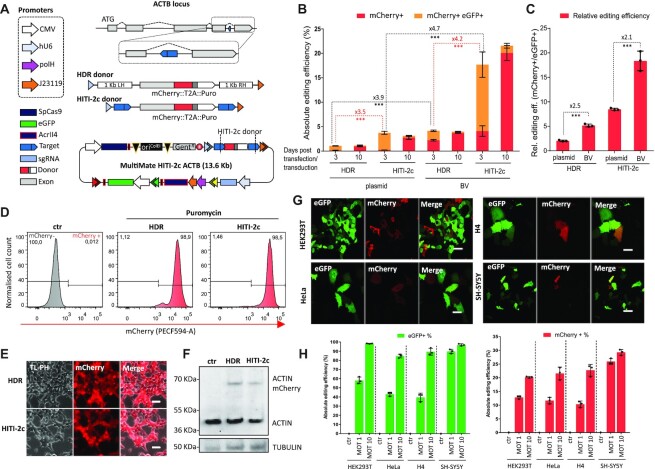
Baculovirus-vectored delivery of complete multicomponent CRISPR/Cas9 toolkits for homology independent targeted integration (HITI) in human cells. (**A**) *ACTB* C-terminal tagging strategy: homologous directed repair (HDR) and homology independent targeted integration (HITI-2c) elements within attL1/attR3 sites (triangles) and MultiMate-HITI-2c ACTB all-in-one DNA circuitry comprising Cas9, HITI-2c donor, sgRNA cassette, eGFP reporter. A module encoding AcrII4 Cas9 inhibitor under control of J23119 and polH promoters ensures vector stability. *ACTB* C-terminal exon is replaced with a synthetic exon, tagged with mCherry::T2A::puromycin. (**B**) Absolute gene editing efficiencies of HEK293T cells transfected or transduced with MultiMate-HDR or MultiMate-HITI-2c BVs in the absence of puromycin selection, at three- and ten-days post-transfection/transduction. (**C**) histogram of relative gene editing efficiencies normalized for transduction/transfection rates at 3 days post transfection/transduction (mCherry + cells %/eGFP + cells %). Histograms represent flow cytometry data. Mean ± s.d. of *n* = 3 independent biological replicates. ***P < 0.001, Student's *t*-test. (**D–F**) HEK293T 21 days after transduction with BV MultiMate HDR or MultiMate HITI-2c BVs after puromycin selection. (**D**) Representative flow-cytometry histograms. (**E**) Widefield microscopy, Scalebar = 20 μm. (**F**) Western blot of total protein extracts. Anti-β-actin antibody was used in top panel with anti-TUBULIN as loading control. (**G**) Confocal images of HEK293T, HeLa, H4 and SH-SY5Y cells 48 hours after transduction with MultiMate-HITI-2c BV. Scalebar is 50 μm. (**H**) Histograms of flow-cytometry data of HEK293T, HeLa, H4 and SH-SY5Y 72 hrs after transduction with MultiMate-HITI-2c VSV-G pseudotyped BVs, multiplicity of transduction (MOT) 1 and 10. Transduction efficiency = % of eGFP + cells; absolute gene editing efficiency = % of Cherry + cells. Mean ± s.d. of *n* = 3 independent biological replicates.

To minimize unpredictable indels and maximise correctly-edited alleles, we sought to analyse and compare HDR and HITI-2c (12) strategies by targeting the intronic β-actin (*ACTB*) locus, introducing a synthetic C-terminal exon fused to mCherry and a self-cleaving peptide (T2A) (51) followed by a puromycin selection cassette (Figure 2A). We counteracted leaky bacterial Cas9 expression and potential leaky Cas9 expression in insect cells by outfitting our DNAs with the Cas9 inhibitor AcrII4 (34) under control of a dual prokaryotic and baculoviral promoter (J23119-polH), preventing vector self-cleavage during bacterial and viral amplifications stages. An additional CMV-eGFP module was added to track transduction efficiency. We next tested plasmid and BV-mediated delivery of MultiMate HDR and HITI-2c constructs in HEK239T cells. The HITI-2c strategy outperformed the HDR based approach (∼4-fold improvement), with a marked gain in editing efficiency when baculovirus transduction was used instead of plasmid transfection (Figure 2B, C, Supplementary Figure S3a, b). The BV backbone was rapidly diluted as we expected (eGFP loss), while mCherry+ cells were stably maintained over time (Figure 2B, C) with absolute gene editing efficiencies reaching ∼5% (HDR) and ∼20% (HITI-2c) (Figure 2b). Notably, when compared to plasmid transfection, baculovirus-vectored delivery increased absolute and relative gene editing efficiency up to ∼4- and ∼2-fold respectively, regardless of the editing approach (Figure 2B, C). To confirm correct gene editing, BV-transduced and plasmid-transfected cells were selected with puromycin and then expanded in the absence of selective pressure, demonstrating stably maintained mCherry expression in close to all (>98%) cells (Figure 2D, E, Supplementary Figure S3c, e). Successful editing was confirmed by PCR genotyping (Supplementary Figure S3d), the expected mCherry subcellular localization (Figure 2E, Supplementary Figure S3e) and the predicted ACTB::mCherry molecular weight (68 kDa) in western blot (Figure 2F, Supplementary Figure S3f). MultiMate-HITI-2c ACTB outperformed HDR editing in all BV transduced cell lines (Supplementary Figure S3g). For optimal efficacy, we prepared VSV-G pseudotyped BVs (21) and transduced HEK293T, HeLa, H4 and SH-SY5Y cells at different multiplicities of transduction (MOT) achieving higher transduction (up to 100%) and editing efficiencies (up to 30%) depending on the cell line (Figure 2H, Supplementary Figure S3). To the best of our knowledge this is the first DNA assembly and delivery platform to enable efficient homology independent targeted integration in mammalian cells using a single all-in-one viral vector.

### Safe-harbour integration of large DNA payloads

**Figure 3.**
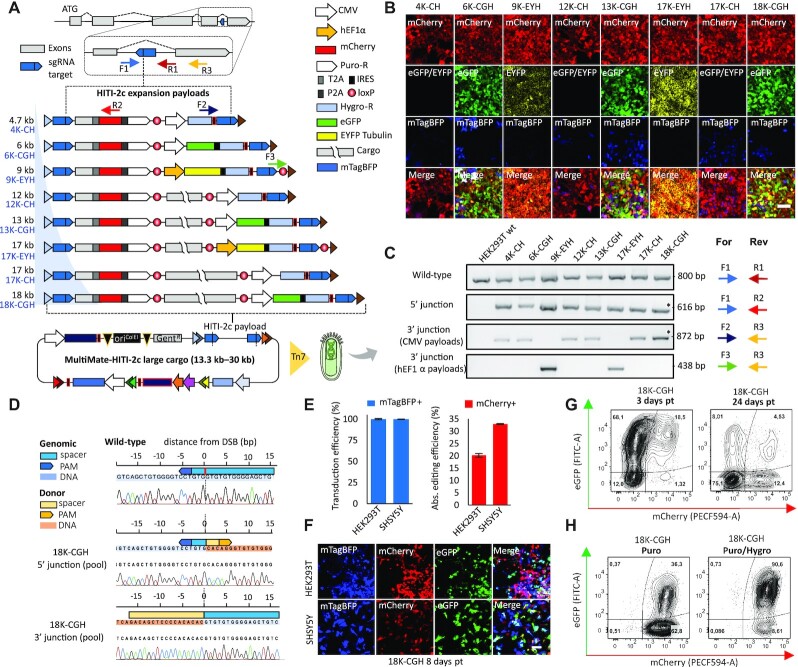
Baculovirus-vectored safe-harbour homology-independent integration of large DNA cargoes in human genomes. (**A**) Safe-harbour HITI-2c strategy, HITI-2c payloads within attL1/attR3 sites (triangles) and MultiMate-HITI-2c ACTB all-in-one plasmid carrying Cas9, HITI-2c payload, sgRNA, AcrII4 and mTagBFP reporter. *ACTB* C-terminal exon is replaced with a synthetic exon, tagged with T2A::mCherry::P2A::puromycin (5′ integration marker), DNA insert ranging from 4.7 to 18 kb, and distinct 3′ integration markers (CMV Hygromycin (CH), CMV eGFP IRES Hygromycin (CGH) or EF1a EYFP-Tubulin IRES Hygromycin (EYH)). (B, C) HEK293T transduced with the indicated MultiMate-HITI-2c BVs after puromycin and hygromycin selection. (**B**) Confocal microscopy. Scalebar, 100 μm. (**C**) PCR genotyping. Oligonucleotide pairs (colour coded arrows) for each PCR are shown, their approximative position is depicted in (A). (**D**) Sanger sequencing of 5′ and 3′ genotyping PCRs (indicated by * in (C)) of HEK293T transduced with MultiMate-HITI-2c 18K-CGH BV. (**E**) Transduction efficiency (left histogram) and absolute gene editing efficiency (right histogram) of HEK293T and SH-SY5Y 72 hours after transduction with MultiMate-HITI-2c 18K-CGH VSV-G pseudotyped BV, derived from flow-cytometry data. Mean ± s.d. of *n* = 3 independent biological replicates. (**F**) Confocal microscopy pictures of HEK293T and SH-SY5Y 8 days after transduction with MultiMate-HITI-2c 18K-CGH VSV-G pseudotyped BVs in the absence of puromycin or hygromycin selection. (**G**) Representative flow-cytometry plots of HEK29T at 3- or 24-days post transduction with MultiMate-HITI-2c 18K-CGH VSV-G pseudotyped BV. (**H**) Representative flow-cytometry plots of HEK29T transduced with MultiMate-HITI-2c 18K-CGH VSV-G pseudotyped BV after puromycin (left) and puromycin/hygromycin selection (right).

Precision docking of large multicomponent DNA circuitry in mammalian genomes remains an impeding challenge for currently available viral delivery systems which are constrained by their intrinsic packaging limitations (AAV: ∼4 kb; LV: ∼8 kb) (19,22). We assessed the aptitude of our system for precision DNA docking exploiting MultiMate-HITI-2c by repurposing the ACTB locus as a safe-harbour docking site for large DNA payloads, equipped with 5′ and 3′ fluorescent and selectable integration markers (Figure 3A and Supplementary Methods). We used a new Cre insertion site to generate a series of HITI-2c payloads ranging from 4.7 kb to 18 kb with mTagBFP as a transduction efficiency reporter, resulting in all-in-one MultiMate plasmids of up to 30 kb (Figure 3A). Transduction with EMBacY BV (33) markedly outcompeted plasmid transfection and editing in HEK293T (Supplementary Figure S4a–d). Upon puromycin selection, cells remained >98% mCherry+ (Supplementary Figure S4e) confirming precise 5′-end integration. We observed silencing of the 3′-end fluorescent marker correlated with cargo size, which we could fully restore by hygromycin selection (Figure 3B, Supplementary Figure S4f). We confirmed correct integration by PCR genotyping and Sanger sequencing at pool level (Figure 3C, D). To confirm integrity of the inserted DNA payload, we FACS sorted mTagBFP-/mCherry+/eGFP+ MultiMate-HITI-2c 18K-CGH transduced HEK293T in absence of any antibiotic treatment. 10 individual clones were cultured and expanded in absence of puromycin/hygromycin. At three months post-transduction, mCherry and eGFP expression were confirmed in all clones (Supplementary Figure S4g). Seven out of nine clones were *bona fide* homozygous KIs (absence of wild-type ACTB amplicon) and all contained intact 5′ and 3′ junctions (Supplementary Figure S4h). Large DNA payload integrity (18 kb) at the ACTB locus was confirmed by 6 spaced PCRs on the intervening DNA cassette. Nine out of 10 clones contained intact DNA payload, while only one clone lost a significant fragment possibly due to recombination (Supplementary Figure S4h–i). We deployed MultiMate-HITI-2c 18K-CGH (30 kb) for baculovirus-vectored delivery with VSV-G pseudotyped BV achieving 100% transduction efficiency in both HEK293T and SH-SY5Y cells giving rise to 20% and 30% absolute genomic insertion efficiency, respectively (Figure 3E). Of note, mCherry expression remained constant over time in the absence of any selective pressure while silencing of 3′ eGFP expression (Figure 3F, G) was again promptly restored by puromycin/hygromycin selection, remaining stable thereafter (Figure 3H). Our results demonstrate that safe-harbour integration of extensive DNA payloads with base-pair precision can be achieved with high efficiency using all-in-one MultiMate-HITI-2c BVs, setting the stage for future large synthetic gene regulatory network engineering in human genomes.

### Rescue of the disease causing R138Q podocin mutation in patient derived podocytes

Podocin is a key membrane scaffolding protein of the kidney podocyte essential for intact glomerular filtration. Mutations in the slit diaphragm protein podocin result in the most common form of monogenic steroid-resistant nephrotic syndrome (SRNS) (52–55. This disease manifests as early childhood onset of proteinuria, fast progression to end-stage renal disease (ESRD) and focal segmental glomerulosclerosis on kidney biopsy (FSGS) with no current treatment option. The most frequent podocin gene mutation in European children is R138Q, causing retention of the misfolded protein in the endoplasmic reticulum (ER) and degradation by the proteasome (40,56). Using temperature-sensitive transgene technology, we have developed human podocyte cell lines from both normal glomeruli (WT ciPods) and glomeruli from a kidney removed due to congenital nephrotic syndrome containing the R138Q mutation of podocin (PM ciPods) (40,57).

**Figure 4.**
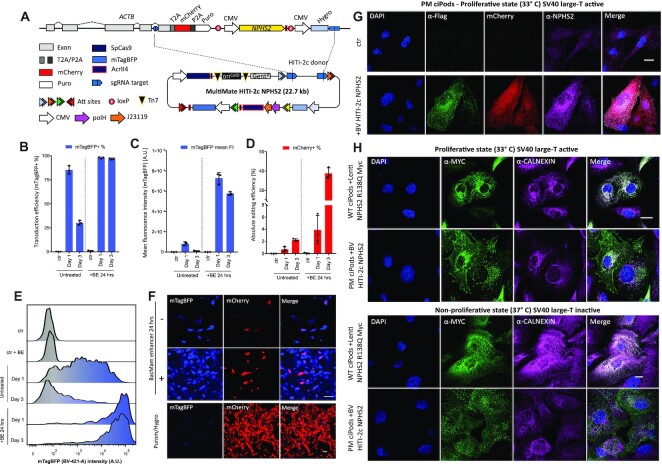
Safe-harbour large cargo integration of NPHS2 into patient-derived R138Q mutant podocin podocytes. (**A**) Safe harbour *ACTB-NPHS2-Myc-Flag* strategy. *ACTB* C-terminal exon is replaced with a P2A mCherry T2A Puro tagged synthetic exon (5′ integration marker), followed by CMV NPHS2-Myc-Flag and CMV Hygro (3′ integration marker). polH VSV-G and CMV mTagBFP are included in vector design to pseudotype and monitor viral transduction, respectively. (B–E) PM ciPods transduced with BV MultiMate HITI-2c NPHS2 in presence/absence of 24 h BacMam enhancer treatment analysed at 24 or 72 h post transduction. (**B**) transduction efficiencies (mTagBFP+ %); (**C**) mTagBFP mean fluorescence intensity levels, (**D**) absolute gene editing efficiencies (mCherry+ %). In (B–E) histograms represents means of flow-cytometry data, error bars are standard deviations of *n* = 3 independent replicates. (**E**) representative flow-cytometry histogram of mTagBFP intensity relative to (C). (**F**) Widefield microscopy images of PM ciPods at 24 h post-transduction in presence/absence of BacMam enhancer (top panel) or 36 days post-transduction following Puromycin/Hygromycin selection (bottom panel). (**G**) Immunofluorescence of unselected PM ciPods transduce with BV MultiMate HITI-2c NPHS2. Successfully edited (mCherry+) cells, display correct expression of NPHS2 through either α-Flag or α-NPHS2 antibodies. DAPI is used to counterstain nuclei. Scalebar = 20 μm. (**H**) Comparison of engineered PM ciPods with WT CiPods overexpressing NPHS2 R138Q under proliferative (33°C, top panel) or non-proliferative (10 days at 37°C, bottom panel) culturing conditions. α-Calnexin labels the endoplasmic reticulum, α-Myc is used to stain NPHS2. Scalebar = 20 μm.

To rescue NPHS2 expression in these cells we sought to deploy our large DNA payload integration strategy to dock a wild-type full-length *NPHS2-Myc-Flag* CDS downstream the *ACTB* locus under the control of a CMV constitutive promoter flanked by T2A::mCherry::P2A::Puro and CMV Hygro acting as 5′ and 3′ selectable markers, respectively (Figure 4A). The resulting construct, (pMm HITI-2c NPHS, 22.6 kb) was tested by transfection in HEK2393T cells. Edited cells (mCherry+) were cultured in presence of Puromycin and Hygromycin for 1 week, showing correct expression and subcellular localization of NPHS2 by immunofluorescence and western blot (Supplementary Figure S4j, k). Next, we transduced patient derived conditionally immortalized podocytes (PM ciPods) (57) with VSV-G pseudotyped BV pMm HITI-2c NPHS2. Although PM ciPods were readily transduced (>70% efficiency) (Figure 4B), transgene expression levels (monitored through mTagBFP expression) were weak and rapidly downregulated within 72 hours (Figure 4B–E), leading to overall low gene editing efficiency (Figure 4D). To counteract this premature silencing, we treated transduced cells with BacMam enhancer (BE), a histone deacetylase inhibitor widely reported to enhance BV-mediated transgene expression. Upon 24 h BE treatment, transduced PM ciPods retained 100% mTagBFP expression up to 72 h post transduction (Figure 4B, C, E), with gene editing efficiencies peaking at ∼40% (Figure 4D). Edited mCherry+ cells were detectable as early as 24 h post-transduction and stably maintained following transient selection with Puromycin/Hygromycin (Figure 4F, Supplementary Figure S4l, m).

Following transduction, NPSH2 expression in mCherry + cells was confirmed by immunofluorescence prior antibiotic selection using either α-Flag and α-NPHS2 antibodies. As expected (56), little to no expression of NPHS2 R138Q could be detected in untransduced PM ciPods (Figure 4G). To better characterize the subcellular localization of NPHS2 in engineered PM ciPods, we sought to compare them with WT ciPods transduced with lentiviral vectors overexpressing NPHS2 R138Q. While NPHS2 R138Q localized entirely at the endoplasmic reticulum, engineered PM Pods displayed NPHS2 localized at the ER and at the plasma membrane, demonstrating functional rescue of NPHS2 trafficking (Figure 4H, top panel). Importantly, transgene expression remained active even after cells were allowed to differentiate by thermo-switching to 37°C, demonstrating correct NPHS2 expression and subcellular localization under non proliferative conditions (Figure 4H, bottom panel). Taken together, these results suggest that large cargo DNA payloads integration using all-in-one BV could be efficiently used to rescue recessive disease-causing genes by integrating a functional copy at a safe harbour locus, in the future ideally under the control of tissue and cell specific promoters.

### Highly efficient multiplexed prime editing

**Figure 5.**
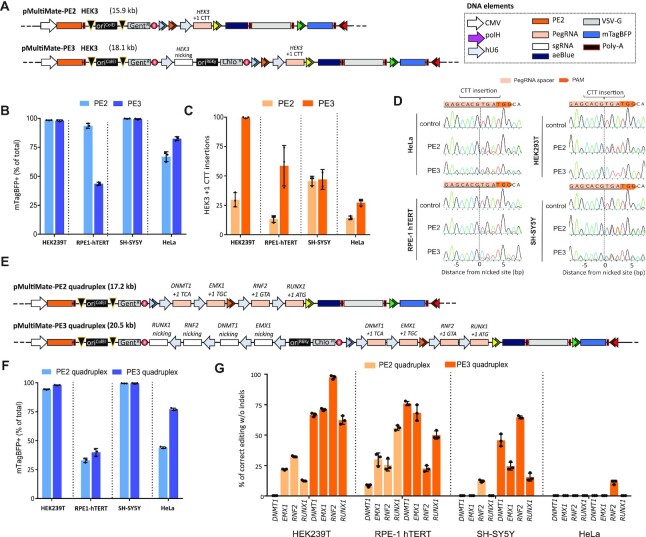
Highly efficient and prime-editing by using MultiMate all-in-one BV. (**A**) Schematic representation of MultiMate-PE2/PE3 *HEK3* for trinucleotide (CTT) insertion. Plasmid DNA size (kb) is indicated. (B–D) HEK293T, RPE-1-hTERT, SH-SY5Y and HeLa transduced with MultiMate-PE2/PE3 HEK3 BVs. (**B**) transduction efficiencies (mTagBFP+ %) at 24 hours post-transduction, flow-cytometry data, error bars represent standard deviation of *n* = 3 independent replicates. (**C**) trinucleotide insertion efficiencies at 6 days post-transduction, sanger sequencing deconvolution data (ICE), error bars are standard deviations of n = 3 independent replicates. (**D**) representative Sanger sequencing alignment of data in (C). (**E**) Schematic representation of MultiMate-PE2/PE3 quadruplex trinucleotide insertion on *DNMT1*, *EMX1*, *RNF2* and *RUNX1*. Plasmid DNA size (kb) is indicated, (F, G) HEK293T, RPE-1-hTERT, SH-SY5Y and HeLa transduced with MultiMate-PE2/PE3 quadruplex BVs, (E) transduction efficiencies (mTagBFP+ %) at 24 h post-transduction, flow-cytometry data, error bars represent standard deviation of *n* = 3 independent replicates. (**F**) trinucleotide insertion efficiencies at 6 days post-transduction, sanger sequencing deconvolution data (ICE), error bars are standard deviations of *n* = 3 independent replicates.

Base editors (BEs) (16,17) and prime editing (PEs) (18) are new additions to the CRISPR toolkit that could potentially correct up to 89% of the human disease-causing mutations in the absence of DNA cleavage (18). PE in particular, can be harnessed to precisely edit genomes with little to no indels production. PE exploits the nickase Cas9-H840A fused to reverse transcriptase from MMLV (PE2) to make a ssDNA copy of the engineered PegRNA at the edited site, allowing for the generation of all possible point mutations, insertions (up to 44 bp) and deletions (up to 80 bp) in the absence of DNA cleavage (18). Prime editing efficiency can be additionally boosted by adding a nicking sgRNA (PE3) which nicks the non-edited strand. Both PE2 and PE3 rely on a Cas9-RT fusion which spans 6.3 kb of DNA (excluding promoter) and codes for a 240 kDa protein. While PE could be virally delivered only through multiple split-intein lentiviral vectors (18) due to cargo limitation, both PE2 and PE3 machinery are entirely within a single baculovirus cargo capacity. We first chose to insert a CTT trinucleotide in the *HEK3* locus using prime editing (18). We therefore assembled MultiMate-PE2 *HEK3* comprising PE2, *HEK3* PegRNA cassette, VSV-G (for pseudotyping), aeBlue chromoprotein (36) (for visual readout of virus titer) and mTagBFP (for transduction tracking) (Figure 5A, Supplementary Figure S5a). MultiMate-PE3 *HEK3* was iteratively assembled by in-vitro CRE fusion (Figure 5A). We next tested transfection-based delivery of MultiMate-PE2 HEK3 in HEK293T which, despite high transfection efficiency, only resulted in 13% CTT insertion at the *HEK3* locus as assessed by Sanger sequencing and deconvolution using ICE (43) (Supplementary Figure S5b, c). Both MultiMate-PE2/PE3 *HEK3* BVs production could be easily monitored with excellent transduction efficiencies in a panel of immortalized human cell lines (Figure 5B and Supplementary Figure S5d, e). Sanger sequencing on *HEK3* locus amplicons from unsorted cells showed correct CTT editing with base-pair precision with correct editing contributions after deconvolution of 15–45% (PE2) and 30–100% (PE3) depending on the cell line, and undetectable indels (Figure 5C, D). While PE3 generally outperformed PE2, BV delivery resulted in almost 100% correct CTT insertion in HEK293T (Figure 5C) after a single viral administration and in absence of any selective pressure. Given the higher susceptibility of HEK293T to BV transduction, we reasoned that this could attributed to a higher transgene expression in this cell line. To investigate this, we serially increased the transduction titer of MultiMate-PE2 *HEK3* and observed a dose-dependent effect boosting correct editing events up to 45%, compared to the 25% of standard viral titers (Figure 5C) without any detectable indels (Supplementary Figure S5f-i) indicating that, despite cell intrinsic factors affecting gene editing outcome, the PE2 expression levels and persistence in target cells are key to enhanced prime editing efficiencies. Since both PE2 and PE3 are well below the cargo capacity of BVs, we sought to explore multiplexed prime editing approaches to simultaneously perform trinucleotide insertions at four different loci. Since MultiMate correctly hosted up to seven CMV promoters (Figure 1B), we chose to exploit individual hU6 promoters/sgRNA cassettes rather than polycistronic sgRNA expression modules (58). MultiMate-PE2 quadruplex was built to simultaneously target *DNMT1*, *EMX1*, *RNF2* and *RUNX1*, while its PE3 counterpart was iteratively assembled by in-vitro CRE fusion with a MultiBac donor harbouring the respective nicking sgRNAs (Figure 5E). Both MultiMate-PE2 and PE3 quadruplex BVs efficiently transduced a panel of immortalized human cell lines (Figure 5E) with similar efficiencies to their single target counterpart (Figure 5B) despite the increase cargo size. Despite overall high editing efficiencies, multiplexed PE2 did not always succeed in simultaneously correcting the four loci, presumably for a rate limiting expression of PE2, now occupied on four different targets (Figure 5G, Supplementary Figure S5j). Efficient and simultaneous targeted PE3-mediated trinucleotide insertions at the four loci could be readily detected in HEK293T (60–100%), RPE-1 (22–76%) and SH-SY5Y (15–64%) but unsatisfactory editing levels in HeLa (Figure 5G, Supplementary Figure S5j), suggesting that transgene expression levels, DNA repair pathways and PegRNA efficiencies weight in more in multiplexed experiments than their single target counterpart. Perhaps due to Sanger sequencing deconvolution detection threshold (43), we were unable to detect low frequencies INDELs events at edited loci, although these are likely to be present as previously described (18). All together, these results provide the first evidence of a single viral delivery vector application for multiplexed prime editing approaches, a valuable tool for multiple gene correction or to establish polygenic disease cell-based models.

### HDR and HITI, but not prime editing, promote undesired backbone integration regardless of the delivery system

Low backbone integration rate is an important safety feature when choosing a suitable delivery system for gene editing interventions. While baculoviruses have been shown to integrate at very low levels in mammalian cells (59), their long-term persistence when coupled with delivery of all-in-one HDR, HITI o prime editors toolkits has not been assessed yet. Indeed, gene editing approaches involving wild-type Cas9 and generation of knock-ins, regardless of the downstream DNA repair pathway used, nature of DNA donor (ssDNA, mini-circle, plasmid), or delivery system (AAVs, plasmid), have inevitably lead to frequent undesired integration of one or more DNA components (14,60–62). While undesired integrations are seemingly unavoidable when using DSBs-producing Cas9, it is reasonable to assume that prime editing approaches, which rely on fusion of nickase-Cas9 with different enzymatic effectors (18), should promote little or no unspecific vector integration.

Since CRISPR toolkits are usually delivered through multiple vectors, fluorescent markers are rarely added on all the DNA or viral species involved, and *ad hoc* sequencing or genotyping experiments are usually designed to unveil the ratio of undesired backbone integration (14,60–62). In contrast, our all-in-one delivery approach allows for real-time monitoring of transfection or transduction marker loss providing a readout of residual backbone integration events.

**Figure 6.**
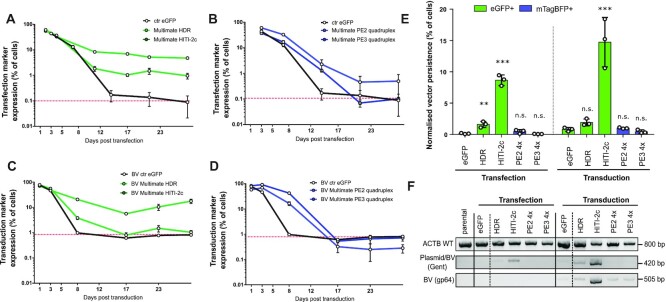
HDR and HITI, but no prime editing, trigger excess backbone genomic integrations. (A–C) 29 days time-course monitoring of transfection and transduction marker in HEK293T transfected (**A, B**) or transduced (**C,D**) with the indicated all-in-one MultiMate constructs harbouring HDR/HITI (A, C) or PE2/3 quadruplex editing toolkits (B, D). A eGFP encoding plasmid or baculovirus, were used as controls for transfections (A, B) and transduction experiments (C, D) respectively. A dashed red line indicates control endpoint detection threshold. Mean with standard deviation of flow-cytometry data of *n* = 3 independent replicates. (**E**) Histogram of backbone associated fluorescence markers persistence at 29 days post-transduction normalised by the initial transfection or transduction efficiency. Mean with standard deviations of *n* = 3 independent replicates. ** P<0.01, ***P < 0.001, n.s.=not significant, Student's t-test. (**F**) Genomic DNA PCRs of HEK293T at 29 days post-transfection/transduction with the indicated constructs. ACTB amplicon was included to check for DNA quality. Gentamycin (Gent) amplicon detects integration events of both plasmids and baculoviruses, gp64 amplicon detects BV integration events only.

To assess backbone integration rates, we monitored fluorescent marker expression in HEK239T (eGFP or mTagBFP) for up to 29 days following transfection or transduction with HDR, HITI-2c, PE2 quadruplex and PE3 quadruplex constructs (Figure 6A–D). While eGFP expressed from a control plasmid was gradually lost over time, MultiMate-HDR and HITI-2c eGFP levels declined up to day 10, afterwards displaying stable eGFP expression in ≈1% and ≈9% of total cell population, respectively (Figure 6A). In contrast, mTagBFP expressed from MultiMate PE2 and PE3 quadruplex plasmid, was gradually lost over time (Figure 6B). While the overall backbone integration rate for a standard eGFP control BV was ≈1%, MultiMate-HDR and HITI-2c BVs resulted in excess backbone integrations as in transfection experiments (Figure 6C, Supplementary Figure S6a). By contrast MultiMate-PE2 and PE3 quadruplex BVs displayed integration rates similar to a control eGFP BV (Figure 6D, Supplementary Figure S6a,b). When normalised for their initial transfection and transduction efficiencies, the backbones of HDR and HITI-2c constructs always integrated at higher rates compared to controls, notwithstanding the delivery system used (Figure 6E), while prime editing constructs residual marker expression levels were indistinguishable from the controls, for both transfection and transduction (Figure 6E). Accordingly, PCR of gentamycin resistance (plasmid and BV specific) or gp64 (BV specific) on genomic DNA, confirmed backbone integration events above threshold for HDR and HITI-2c, but not for PE2 and PE3 quadruplex constructs (Figure 6F), regardless the delivery system.

Cells transduced with MultiMate-HDR BVs resulted in excess eGFP integration mostly within the mCherry+ cells, while MultiMate-HITI-2c transduced cells displayed increased backbone integration rates in both mCherry + and mCherry- cells (Supplementary Figure S6a), most likely as result of donor excision through Cas9 activity. HDR and HITI-2c puromycin selected cells, revealed overall high levels of backbone integration, independently from the delivery method or the editing approach (Supplementary Figure 6Sc–e). In particular, within edited cells, both HDR and HITI-2c had similar levels of backbone integration upon transfection or transduction (25–30%), with BV-mediated delivery partially mitigating the backbone integration rates only for HDR (Supplementary Figure S6e).

Finally, we isolated eGFP- and eGFP+ clones from HEK293T transduced with BV MultiMate-HITI-2c and puromycin selected. Two out of three eGFP- clones where homozygous KIs (absence of WT ACTB band), while all the eGFP + clones were heterozygous. 5′ integration was correctly detected in all clones, and BV integrations were only detectable in eGFP + clones (Supplementary Figure S6f), confirming that eGFP late expression is a *bona fide* marker for backbone integration. In contrast, no BV integration events were detectable at clonal level in BV MultiMate PE3 quadruplex transduced cells, again confirming that prime editors do not promote undesired backbone integration events.

Overall, we confirmed that basal baculovirus integration levels do not exceed 1%, rendering them well suited to deliver different CRISPR technologies. Backbone integration levels are however dependent on the editing technology of choice, and both HDR and HITI-2c, which rely on DSBs-producing Cas9 variants and DNA donors, result in exceedingly high levels of backbone integration regardless the delivery system. By contrast prime editors promote little to no backbone integration by either transfection or baculovirus-mediated transduction.

## DISCUSSION

The rapid evolution of CRISPR approaches for precise genome-engineering precipitated an urgent need for next-generation viral delivery vector systems with superior DNA cargo capacity, efficiency and safety as compared to the state-of-the-art (20). The limited packaging capacity of currently dominating viral vectors (AAVs, LVs) constrains precise genome editing interventions (19,22), requiring co-transduction with multiple viruses (12,18) or implementation of smaller Cas variants (CasX, CasΦ) (63,64) even for simple Cas/sgRNA approaches. While these technologies can cope, although suboptimal, with delivery into cultured cells, the viral titer required for successful co-transduction *in vivo* will likely dampen gene editing efficiency. Additionally, the size of the genome editing intervention in gene replacement experiments, cannot physically be extended beyond the cargo capacity of the vector of choice.

We demonstrated here the efficacy of our baculovirus-vectored approach to overcome this limitation and tackle the CRISPR delivery challenge. We optimized DNA assembly (MultiMate) to facilitate vector construction and improve BV manufacturing. We implemented homology independent targeted integration (HITI) for precise insertion achieving efficient precision C-terminal tagging in the *ACTB* locus, markedly outcompeting HDR efficiency. Using our approach, we demonstrated precision safe-harbour integration of large multicomponent DNA payloads with outstanding efficiencies and immediate potential for synthetic biology applications. We show that correct integration, however, is not sufficient to ensure stable expression of all the integrated components, likely due to transgene silencing events. Reassuringly, we demonstrate that usage of 5′ and 3′ junctions-linked selectable markers (fluorescent proteins or antibiotic resistance cassettes) can efficiently select for phenotypically and genotypically correctly edited cells. Synthetic biology applications in primary cell lines however, will require testing of alternative promoters (65), different safe-harbour integration sites (66) and incorporation of DNA insulator elements (67,68) to counteract gene silencing and ensure functionality of multicomponent synthetic circuits in absence of selection. There is no indication that we reached the cargo limit of baculovirus-vectored delivery. In fact, given the wide variation in size of naturally occurring baculoviruses (69) we expect that delivery of DNA cargos exceeding 100 kb will likely be feasible, enabling insertion of entire metabolic pathways and gene regulatory networks in safe-harbour sites or elsewhere in genomes. The large DNA payload integration strategy could in future be used to rescue recessive disease-causing genes by supplementing cells with wild-type CDS integrated at a safe harbour site. In this regard, we provided proof-of-concept rescue of NPHS2 expression in SRNS patient derived podocytes (57). By installing a wild-type NPHS2 copy in the ACTB locus, we rescued NPHS2 trafficking phenotype and expression levels, theoretically demonstrating the feasibility of this approach for any recessive disease-causing gene. While we chose to install a CMV driven NPHS2 expression module, tissue and cell specific promoters will need to be tested and implemented in future to ensure transgene expression at physiologically relevant levels only within specific cell types. While *in vivo* applications remain theoretical, this approach could be applied to *ex vivo* modification of stem or primary cells, for instance enabling more affordable CAR-T manufacturing.

Importantly, we demonstrate the utility of our approach for seamless search-and-replace gene editing by implementing recently developed prime editing (PE) technology (18). Particularly PE, is considered safer when compared to HDR or HITI-2c, which rely on DNA cleavage. Using baculovirus-vectored delivery, we achieved highly efficient PE3-mediated trinucleotide insertion (CTT) in the *HEK3* locus with up to 100% efficiency in the absence of detectable indels by using a single viral vector, in contrast to co-transduction with four lentivectors as previously reported (18). Additionally, we provide evidence of the feasibility of multiplexed prime editing approach to simultaneously insert trinucleotide insertions with high efficiencies (up to 60–97%) and, again, using a single viral vector with up to 8 hU6 PegRNA/sgRNA expression cassettes. We foresee that updated prime editing variants as PEmax (70) or more complex seamless drag-and-drop approaches (71) could be readily implemented for baculoviral-mediated delivery in future, further expanding the range of genome engineering applications.

We further demonstrate here that baculovirus vectors are inherently safe and do integrate in mammalian cells at rates close or below 1%. However, we have found that editing approaches which rely on DSBs Cas9 variants, inevitably lead to excess backbone integrations, regardless of the delivery system. In our hands, up to 25%-30% of edited cells displayed long term retention of transfection/transduction marker in HDR and HITI-2c experiments, suggesting plasmid and viral backbone integrations triggered by DSBs in addition to correct edits. Accordingly, various degrees of backbone integration have been reported with HDR and HITI approaches, notwithstanding the delivery system or the nature of the DNA donor (14,60–62). In marked contrast, prime editing approaches, which rely on nickase Cas9 variants, could be safely delivered by either plasmid or baculoviruses, and displayed similar integration rates to non-editing constructs. While these two classes of gene editing enable two distinct sets of interventions (gene replacement for HDR and HITI and gene correction for PE), it appears evident that HDR and HITI are best suited to synthetic biology or *ex vivo* gene editing, while PE applications could be more impactful on the short term for *in vivo* applications. A new class of editors that combines the safety of prime editing with the size of the intervention range of HDR and HITI has yet to emerge, although hybrid approaches combining prime editing and integrase technologies such as twinPE and PASTE (71,72), could provide enhanced safety and reduced backbone integration, while still allowing for large DNA editing. Although requiring additional modules, both technologies could be easily accommodated on a single baculovirus, while delivery through other viral or non-viral delivery system will prove even more challenging.

Taken together, our results establish baculovirus as a vector of choice for precision engineering of large DNA cargoes and single/multiplexed prime editing in mammalian cells, and we anticipate baculovirus-enabled large-scale genome interventions, even combining safe-harbour integration with concomitant, if needed multiplexed, base or prime editing strategies, enabling complex synthetic biology approaches and, in future, *ex vivo* gene editing in clinically relevant cells.

Several roadblocks, which we recently reviewed (73), still stand in the way of *bona fide* baculovirus applications *in vivo*. Compared to more common viral vectors, only a handful of reports exist for baculovirus mediated gene delivery *in vivo* (22,74–76), none of them including clinical trials in humans. Human serum complement, for instance, is known to inactivate gp-64 enveloped baculoviruses *in vitro*. This has been addressed by engineering modified envelopes to overcome this limitation. While VSV-G is able to partially shield from mouse serum complement *in vivo* (74,75), it provides little to no protection against rat and human serum *in vitro* (75). Pseudotyping with decay accelerating factor (DAF) however, has been shown to effectively shield virions from human serum complement (77,78). On this note, complement shielding has been achieved also by functionalising BVs with magnetic nanoparticles, enabling efficient CRISPR editing *in vivo* in mice (76). In addition, promoter silencing (79,80) and intracellular innate immunity driven by the cGAS/STING signalling pathway inactivate baculovirus in cultured mammalian cells (81,82), representing a challenge for both *ex vitro* and *in vivo* gene delivery and editing approaches. Histone deacetylase inhibitors (79,80) and small molecule STING antagonist (83) have been used with varying degree of success to counteract these challenges in cultured cells. While these molecules could theoretically pave the way for baculovirus mediated gene editing *ex vivo* in the future, more work will be needed to engineer baculoviral vectors suitable for efficient gene delivery *in vivo*. Contrary to other viral vectors however, the large cargo capacity of baculovirus will easily enable the incorporation of transduction helper modules, pending the elucidation of the molecular mechanisms underlying their inactivation in mammalian cells.

## DATA AVAILABILITY

All plasmid sequences are provided in Supplementary Table S1. MultiMate-CellCycle, MultiMate-Rainbow and MultiMate-HITI-2c ACTB reagents will be made available for distribution by Addgene. Raw flow-cytometry data have been deposited under the following DOI: https://doi.org/10.6084/m9.figshare.20110364.v1. All other reagents are available from the authors upon reasonable request.

## Supplementary Material

gkac587_Supplemental_Files

## Notes

Present address: Martin Pelosse, EMBL Grenoble, Eukaryotic expression facility, 71 Avenue des Martyrs CS 90181, 38042, Grenoble Cedex 9, France.
