# Supplementary material for: Highly efficient CRISPR-mediated large DNA docking and multiplexed prime editing using a single baculovirus
Source: Nucleic Acids Res. 2022 Jul 8;50(13):7783–99. doi: 10.1093/nar/gkac587 (PMC9303279; doi:10.1093/nar/gkac587)
Supplement: gkac587_Supplemental_Files [file gkac587_supplemental_files.zip › Aulicino et al. Supplementary Data.pdf]

Supplementary Data

Highly efficient CRISPR-mediated large DNA docking and multiplexed prime editing using a single baculovirus

Francesco Aulicino[1]*, Martin Pelosse[1], Christine Toelzer[1], Julien Capin[1], Erwin Ilegems[2], Parisa Meysami[1], Ruth Rollarson[3], Per-Olof Berggren[2], Mark Simon Dillingham[1], Christiane Schaffitzel[1], Moin A. Saleem[3], Gavin I. Welsh[3] and Imre Berger[1,4]*

[1] BrisSynBio Bristol Synthetic Biology Centre, Biomedical Sciences, School of Biochemistry, 1 Tankard's Close, University of Bristol, Bristol BS8 1TD, United Kingdom
[2] The Rolf Luft Research Center for Diabetes and Endocrinology, Karolinska Institutet, SE-171 76 Stockholm, Sweden
[3] 2 Bristol Renal, Bristol Medical School, Dorothy Hodgkin Building, Whitson street, Bristol BS1 3NY, United Kingdom
[4] Max Planck Bristol Centre for Minimal Biology, School of Chemistry, University of Bristol, Cantock's Close, Bristol BS8 1TS, United Kingdom

* To whom correspondence should be addressed. Tel: +44 117 394 1857; Email: imre.berger@bristol.ac.uk
Correspondence may also be addressed to Francesco Aulicino. Email: francesco.aulicino@bristol.ac.uk

Present Address: Martin Pelosse, EMBL Grenoble, Eukaryotic expression facility, 71 Avenue des Martyrs CS 90181, 38042, Grenoble Cedex 9, France

List of contents

Supplementary Data **1**

Highly efficient CRISPR-mediated large DNA docking and multiplexed prime editing using a single baculovirus **1**

Supplementary Figures Legends **3**

Supplementary Fig. S1. MultiMate assembly protocol and validation. 3

Supplementary Fig. S2. MultiMate assembly for baculovirus-vectored delivery of large multifunctional DNA in human cells........ 4

Supplementary Fig. S3. Baculovirus-vectored delivery of complete multicomponent CRISPR/Cas9 toolkits for homology independent targeted integration (HITI) in human cells, continued........ 5

Supplementary Fig. S4. Baculovirus-vectored safe-harbour homology-independent integration of large DNA cargoes in human genomes, continued. 6

Supplementary Fig. S5. Highly efficient single and multiplexed prime-editing by using MultiMate all-in-one BV, continued 7

Supplementary Fig. S6. HDR and HITI, but no prime editing, trigger excess backbone genomic integrations, continued 8

Supplementary Methods **9**

Design and implementation of MultiMate DNA assembly........ 9

MultiMate-HITI-2c for large DNA safe-harbour integration........ 10

Supplementary Tables **11**

Supplementary Table S1. Vectors list and assembly details (provided as Excel file) 11

Supplementary Table S2. Gene editing outcomes, sgRNA sequences and DNA oligos (provided as Excel file)........ 11

Supplementary Table S3. attL/R sequences and attB recombination products after LR reaction........ 12

Supplementary Table S4. Cell lines, origin and media composition. 13

Supplementary Table S5. List of antibodies used........ 14

Supplementary Equation **14**

Supplementary Equation 1. Baculovirus end-point dilution titration. 14

Supplementary Movie **14**

Supplementary Movie S1. MultiMate-CellCycle BV transduced HeLa (provided as movie file) 14

Supplementary Figures Legends

Supplementary Fig. S1. MultiMate assembly protocol and validation. **a**, DNA elements are pasted into four distinct ENTR plasmid modules in between attL/R recombination sites (coloured triangles). **b**, One DEST module and four ENTR modules are assembled by LR recombination *in vitro*. DEST plasmids are adapted from MultiBac Acceptor/Donor suite (*26,33*). In pMMACE DEST, the homing cassette is attR1-Ccdb-Chlo^{R}-attR2, in pMMDS DEST attR1-Ccdb-Ori^{ColE1}-attR2. **c**, After LR reaction, pMMACE DEST and pMMDS DEST are fused by Cre-recombination. **d**, MultiBac donor plasmids and pMMD DEST vectors can be iteratively added by Cre recombination, barcoded by resistance makers[27]. **e**, MultiMate plasmid is shuttled into the MultiBac BV by Tn7-mediated recombination in *E. Coli*. MultiMate and MultiBac BVs can be further functionalised by *in vitro* Cre-mediated recombination (d-e). **f**, Plasmid size, number of modules and cargo to prokaryotic backbone DNA ratio (green and grey bars, respectively) for hypothetical 1.5 kb expression cassettes. **g**, Assembly strategy for MultiMate plasmid encoding chaperonin CCTs. CCT subunits were pasted into pMMK ENTR plasmid modules comprising baculoviral promoters (polH, p10), assembled by LR reaction into pMMACE DEST and pMMDS DEST, fused by Cre and loaded on EMBacY (*33*) BV. Expression of MultiMate-CCTs with EMBacY in Sf21 insect cells produces complete CCT/TriC chaperonin. **h**, Western blot of infected Sf21 cells, uninfected cells as control (ctr) with CCT subunit specific antibodies. Anti-His was used to detect His-CCTα. **i**, SDS-PAGE analysis of purified CCT/TriC chaperonin complex. **j**, Negative-stain EM (top) and 2D-class averages (bottom) of purified CCT/TriC. Scalebar, 100 nm.

Supplementary Fig. S2. MultiMate assembly for baculovirus-vectored delivery of large multifunctional DNA in human cells. **a**, MultiMate-Rainbow assembly in a schematic view. Individual modules in pMMACE, pMMDS DEST and pMMK ENTR as shown (upper panel). Two LR reactions resulted in MultiMate-5-colours and pMMDS-2-colours (middle panel), fused by Cre-mediated recombination *in vitro* to produce MultiMate-Rainbow. **b**, Restriction mapping of five clones each of MultiMate-5-colours (left) pMMDS-2-colours (middle) and Cre-recombined MultiMate-Rainbow (right) evidences robust assembly. **c**, Confocal images of HEK293T, HeLa, H4 and SH-SY5Y cells 48 hours after transduction with MultiMate-5-colours VSV-G pseudotyped BV[20]. Scalebar, 20 µm. **d**, Schematic representation of MultiMate-CellCycle assembly. Individual modules were pasted in pMMACE and pMMK ENTR plasmids (upper panel). **e**, Restriction mapping of five clones each after LR recombination. **f**, Confocal microscopy of HEK293T, SH-SY5Y, HeLa and H4 cells at 48 hours after transduction with MutiMate-CellCycle VSV-G pseudotyped BV. Scalebar, 100 µm.

Supplementary Fig. S3. Baculovirus-vectored delivery of complete multicomponent CRISPR/Cas9 toolkits for homology independent targeted integration (HITI) in human cells, continued. a-b, Representative flow-cytometry plots of HEK293T cells transfected with MultiMate-HDR or HITI-2c all-in-one plasmids (a) or transduced with baculovirus (BV) (b) in the absence of puromycin selection at three- or ten-days post transfection/transduction. **c**, Representative flow-cytometry histograms of HEK293T transfected with MultiMate-HDR or MultiMate-HITI-2c after puromycin selection. **d**, Genotype PCR of HEK293T transfected (upper panel) or transduced (lower panel) with MultiMate-HDR or MultiMate-HITI-2c after puromycin selection. **e**, Widefield microscopy images of live HEK293T transfected with MultiMate-HDR or MultiMate-HITI-2c plasmids after puromycin selection. Scalebar, 100 µm. **f**, Western blot of total protein extracts from puromycin selected HEK293T transfected with MultiMate-HDR, MultiMate-HITI-2c and untransfected HEK293T as a control (ctr). Anti-β-actin antibody was used in top panel, with anti-TUBULIN as a loading control. **g**, Representative confocal images of HEK293T, HeLa, H4 and SH-SY5Y human cells 72 hours after transduction with MultiMate-HDR or HITI-2c BV. Scalebar, 50 µm. **h**, Representative flow-cytometry plots of HEK293T, HeLa, H4 and SH-SY5Y cells at three-days post transduction with MultiMate-HITI-2c BV, untransduced parental cell lines as a control (ctr).

Supplementary Fig. S4. Baculovirus-vectored safe-harbour homology-independent integration of large DNA cargoes in human genomes, continued. a-b, Delivery efficiencies of indicated MultiMate-HITI-2c plasmids by transfection (**a**) or low-titer BV transduction (b) in HEK293T at three-days post transfection/transduction. **c-d**, Absolute gene editing efficiencies of HEK293T at three-days post transfection (c) or low-titer BV transduction (d) with the indicated MultiMate-HITI-2c plasmids. Mean ± s.d. of n = 3 independent biological replicates. Dots and triangles represent total MultiMate plasmid and HITI-2c payload DNA sizes, respectively. **e-f**, Representative flow-cytometry histograms of HEK293T transduced with the indicated MultiMate-HITI-2c BVs and selected with puromycin or puromycin/hygromycin for mCherry expression (**e**) or eGFP/EYFP expression (f). **g-i,** Analysis of mTagBFP-/mCherry+/eGFP+ clonal cell lines isolated through FACS-sorting from HEK293T transduced with MultiMate-HITI-2c-18K-CGH and expanded in absence of antibiotics. g, widefield microscopy images, scalebar = 50 µm. h, genomic DNA PCRs against 5' and 3' junctions and an additional 6 amplicons spanning the large DNA payload; **i**, schematic representation of DNA cargo integrity within its genomic context inferred from PCRs data in (h). **j-k**, HEK293T transfected with pMm HITI-2c NPHS2 following Puromycin/Hygromycin selection. **j,** Immunofluorescence of NPHS2-Myc, DAPI is used to counterstain nuclei, scalebar=20 µm. **k**, western blot on total protein extract using anti-NPHS2, anti-Flag and anti-TUBULIN as loading control. **l**, Representative flow-cytometry plots of PM podocytes transduced with BV pMm HITI-2c NPHS2 and treated with BacMam enhancer for 24 hours. Cells were analysed at 24 and 72 hours post-transduction. mTagBFP is the transduction reporter, mCherry is the gene editing efficiency reporter. **m**, Representative flow-cytometry plots of PM podocytes transduced with BV pMm HITI-2c NPHS2 following Puromycin/Hygromycin selection.

Supplementary Fig. S5. Highly efficient single and multiplexed prime-editing by using MultiMate all-in-one BV, continued a, Restriction mapping of five randomly picked MultiMate-PE2 clones. **b**, Confocal microscopy images of HEK293T two-days after transfection with MultiMate-PE2 plasmid. **c**, Representative genotype PCR Sanger sequencing of HEK293T four-days after transfection with MultiMate-PE2 and relative sequences contribution after Sanger sequencing deconvolution (ICE). Mean ± s.d. of n = 3 independent biological replicates. **d**, Visual summary of BV amplification. V0: widefield images of Sf21 cells in 6-well plates after initial transfection with MultiMate-PE2 BV DNA and control cells (untransfected). All transfected cells show VSV-G induced syncytia. V2: aeBlue expression in suspension culture (upper panel) and centrifuged pellets (lower panel) of Sf21 cells during amplification of MultiMate-PE2 BVs. **e**, Confocal microscopy images of HeLa, RPE-1 hTERT, HEK293T and SH-SY5Y at 24-hours post transduction with MultiMate-PE2 BV. **f**, Representative flow-cytometry histograms of mTagBFP expression in HEK293T at 24-hours post transduction. Dotted lines represent gating. **g**, Transduction efficiencies of HEK293T at 24-hours post-transduction with the indicated dilutions of MultiMate-PE2 BV. Mean ± s.d. of n = 5 independent biological replicates. **h**, Sanger sequencing comparisons of of *HEK3* locus in HEK293T six days after transduction with the indicated amounts of MultiMate-PE2 BV. **i**, Percentage of correct editing (CTT insertion) in HEK293T six days post transduction with the indicated amounts of MultiMate-PE2 BV. Data are derived from Sanger sequencing deconvolution (ICE), no indels were detected in any of the conditions. Mean ± s.d. of n = 3 independent biological replicates. **j**, Representative Sanger sequencing trace alignments of parental (ctr) and transduced cell lines with PE2 quadruplex or PE3 quadruplex baculovirus. Rows: genes, Columns: cell lines.

Supplementary Fig. S6. HDR and HITI, but no prime editing, trigger excess backbone genomic integrations, continued

a-b, Representative flow-cytometry plots of HEK293T at 29 days post-transduction with the indicated baculoviruses, **a** mCherry/eGFP plots for untransduced (parental), control (BV eGFP) and MultiMate HDR and HITI-2c transduced cells, **b** eGFP/mCherry plots for untransduced (parental), and MultiMate PE2/PE3 quadruplex transduced cells. **c-d**, HEK293T transfected or transduced with MultiMate HDR (**c**) or HITI-2c (**d**) constructs after puromycin selection analysed at 1 month post-transfection/transduction. **e**, histogram of flow cytometry data in (c,d), error bars are standard deviations of n=3 independent replicates. **f-g**, genomic DNA PCRs to detect viral backbone integrations in clonal cell lines isolated from HEK293T transduced with MultiMate-HITI-2c ACTB BV and puromycin selected (**f**) or MultiMate-PE3 quadruplex BV (**g**). A gentamycin (Gent) amplicon was used to detect viral backbone integrations.

Supplementary Methods

Design and implementation of MultiMate DNA assembly

We first engineered four ENTR vectors (pMMK ENTR 1-4) in which individual DNA modules are flanked by specific LR attachment sites (attL/R). We named these vectors pMMK ENTR 1-4 (**Fig.1a, Fig. S1a)**, reflecting flanking attL/R sites and the position of the enclosed modules in the final vector (attL1/R3 (1), attL3/R4 (2), attL4/R5 (3) and attL5/R2 (4)) (**Fig.1a, Fig. S1a**). Next, we repurposed MultiBac acceptors (pACE) and donors (pMD)[32] as MultiSite Gateway DEST vectors. To generate pMMACE DEST, pACE was equipped with *Ccdb* toxin and Chloramphenicol resistance cassettes between attR1/attR2 sites (Fig 1a, S1b). pMMACE DEST vectors can only be propagated in suitable *E. Coli* strains harbouring the *gyrA462* mutation or expressing the *Ccda* anti-toxin (e.g *Ccdb* survival cells or *E. Coli* F^+). Conversely, MultiBac donors rely on R6Kγ[32,44] replication origin and can only be propagated in *Pir+* cells but no *E. Coli* strains that feature both R6Kγ replication origin (*Pir+*) and *Ccdb* resistance (*Ccda+* or *gyrA462+*) have been engineered to date. To overcome this problem, pMMDS DEST was generated by replacing the Chloramphenicol cassette with a *ColE1* origin of replication, and propagated in standard *Ccdb* survival *E.Coli* (**Fig.1a, Fig. S1b**). Upon successful LR recombination between one DEST and four pMgK ENTR vectors, the *Ccdb* toxin is lost, and the resulting pMMACE DEST or pMMDS DEST assembled vectors can be transformed into conventional (Top10, DH5α) or Pir+ *E.Coli* strains respectively (**Fig.1a, Fig. S1b**). Finally, loaded pMMACE and pMMDS can be fused using Cre-mediated recombination (**Fig. S1c**), iteratively complemented with additional donors (**Fig. S1c**), or directly loaded into standard or pre-functionalised MultiBac (**Fig. S1d-e**) for BV production as previously described[19,32,44]. Notably, MultiMate allows for a substantial reduction of subsidiary increasing cargo to backbone ratio and reducing the amount of repetitive DNA elements in the final BV (**Fig. S1f**).

MultiMate-HITI-2c for large DNA safe-harbour integration

MultiMate-HITI-2c for large cargo integration (**Fig.3a**) was repurposed from MultiMate-HITI-2c-hACTB (**Fig.2a**). The loxP site from pMMACE Cas9 DEST was relocated on three different HITI-2c donor plasmids in between 5' and 3' sgRNA target sites (Fig.3a). All HITI-2c donor plasmids were equipped with 5' (T2A::mCherry::P2A::Puro) and 3' selection markers (either CMV Hygro (CH), CMV eGFP IRES Hygro (CGH), or EF1α EYFP::Tubulin IRES Hygro (EGH)) flanking a loxP site which served to conveniently extend the size of the intervening payload by CRE-mediated recombination. Plasmids name abbreviations indicate payload size and 3' marker (e.g. 18K-CGH is 18 kb payload with CMV eGFP IRES Hygro 3' integration marker). For safe-harbour integration of wild-type NPHS2, pMDC CMV wtNPHS2 Myc Flag was fused to MultiMate-HITI-2c 4K-CH by CRE-mediated recombination *in vitro* to generate MultiMate-HITI-2c NPHS2. Additional assembly information is provided in **Table S1**.

Supplementary Tables

Supplementary Table S1. Vectors list and assembly details (provided as Excel file)

Supplementary Table S2. Gene editing outcomes, sgRNA sequences and DNA oligos (provided as Excel file)

Supplementary Table S3. attL/R sequences and attB recombination products after LR reaction.

attR/attB sequences

Name	Sequence (5'-3')
attR1	Acaagtttgtacaaaaaagctgaacgagaaacgtaaaatgatataaatatcaatatattaaattagattttgcataaaaaacagact acataatactgtaaaacacaacatatccagtcactatg
r_attR2	Catagtgactggatatgttgtgttttacagtattatgtagtctgttttttatgcaaaatctaatttaatatattgatatttatatcattttacg tttctcgttcagctttcttgtacaaagtggt
attL1	Caaataatgattttattttgactgatagtgacctgttcgttgcaacaaattgataagcaatgcttttttataatgccaactttgtacaaaa aagcaggct
r_attL2	Agacagctttcttgtacaaagttggcattataagaaagcattgcttatcaatttgttgcaacgaacaggtcactatcagtcaaaataaa atcattatttg
attL3	AAATAATGATTTTATTTTGACTGATAGTGACCTGTTCGTTGCAACAAATTGATGAGCAATGCTTTTTTAT AATGCCAACTTTGTATAATAAAGTTG
attR3	caactttgtataataaagttgaacgagaaacgtaaaatgatataaatatcaatatattaaattagattttgcataaaaaacagactac ataatactgtaaaacacaacatatccagtcactatg
attL4	AAATAATGATTTTATTTTGACTGATAGTGACCTGTTCGTTGCAACAAATTGATAAGCAATGCTTTCTTAT AATGCCAACTTTGTATAGAAAAGTTG
attR4	CCAACTTTGTATAGAAAAGTTGAACGAGAAACGTAAAATGATATAAATATCAATATATTAAATTAGATT TTGCATAAAAAACAGACTACATAATACTGTAAAACACAACATATCCAGTCACTATG
attL5	aaataatgattttattttgactgatagtgacctgttcgttgcaacaaattgatgagcaatgcttttttataatgccaactttgtatacaaaa gttg

LR recombination products

L/R	attB product	Sequence (5'-3')
attL1/attR1	attB1	ACAAGTTTGTACAAAAAAGCAGGCT
r_attL2/r_attR2	r_attB2	CAGCTTTCTTGTACAAAGTGG
attL3/attR3	attB3	CAACTTTGTATAATAAAGTTG
attL4/attR4	attB4	CAACTTTGTATAGAAAAGTTG
attL5/attR5	attB5	CAACTTTGTATACAAAAGTTG

Supplementary Table S4. Cell lines, origin and media composition.

Species and tissue of origin are listed for each cell lines. Reagent manufacturers and catalogues numbers are listed.

Cell line	Source	Media recipe (500 ml)
HEK293T<br>(*H.Sapiens*)<br>Embryonic kidney	ATCC #CRL-3216	• 445 ml DMEM (Thermo Fisher, # 41965039)<br>• 50 ml FBS (Thermo Fisher, # 10270106)<br>• 5 ml 10000 U/ml Penicillin/ 10000 µg/ml Streptomycin (Gibco, #15140122)
HeLa<br>(*H.Sapiens*)<br>Cervical cancer	ATCC #CCL-2	• 445 ml DMEM (Thermo Fisher, # 41965039)<br>• 50 ml FBS (Thermo Fisher, # 10270106)<br>• 5 ml 10000 U/ml Penicillin/ 10000 µg/ml Streptomycin (Gibco, #15140122)
H4<br>(*H.Sapiens*)<br>Neuroglioma	ATCC #HTB-148	• 445 ml DMEM (Thermo Fisher, # 41965039)<br>• 50 ml FBS (Thermo Fisher, # 10270106)<br>• 5 ml 10000 U/ml Penicillin/ 10000 µg/ml Streptomycin (Gibco, #15140122)
RPE-1 hTERT<br>(*H.Sapiens*)<br>Retinal pigmented epithelia (immortalised)	ATCC #CRL-4000	• DMEM/F12 (Thermo Fisher # 11320033)<br>• 10% FBS (Thermo Fisher, # 10270106)<br>• 5 ml 10000 U/ml Penicillin/ 10000 µg/ml Streptomycin (Gibco, #15140122)
SH-SY5Y<br>(H.Sapiens)<br>Neuroblastoma	ATCC #CRL-2266	• 222.5 ml EMEM (Gibco #670086)<br>• 222.5 ml Ham's F12 (Gibco #11765054)<br>• 50 ml FBS (Thermo Fisher, # 10270106)<br>• 5 ml 10000 U/ml Penicillin/ 10000 µg/ml Streptomycin (Gibco, #15140122)
WT ciPods Lenti NPHS2 R138Q<br>*(H. Sapiens)*	Described in Harris, Saleem M.A. et al. (2002)	• 440 ml RPMI (Sigma, #R8758-500ML)<br>• 50 ml FBS (Thermo Fisher, # 10270106)<br>• 5 ml 10000 U/ml Penicillin/ 10000 µg/ml Streptomycin (Gibco, #15140122)<br>• 5 ml Insulin/Transferrin (Gibco, #41400-45)
PM ciPods R138Q<br>*(H. Sapiens)*	Described in Harris, Saleem M.A. et al. (2002)	• 440 ml RPMI (Sigma, #R8758-500ML)<br>• 50 ml FBS (Thermo Fisher, # 10270106)<br>• 5 ml 10000 U/ml Penicillin/ 10000 µg/ml Streptomycin (Gibco, #15140122)<br>• 5 ml Insulin/Transferrin (Gibco, #41400-45)
Sf21<br>(S. Frugiperda)<br>Ovary cell line	Thermo Fisher #11497013	• Sf-900 II SFM (Themo Fisher #10902096)

Supplementary Table S5. List of antibodies used.

Manufacturers, catalogues numbers and working dilution are listed.

Antibody	Vendor #catalog number	Working dilution
anti-β Actin (HRP)	abcam #ab49900	1:1000
anti-Tubulin	Santa Cruz #sc-53030	1:1000
Goat Anti-Rat (HRP)	abcam #ab49900	1:2000
Anti-6X His tag antibody (HRP)	abcam # ab1269	1:1000
Anti CCTβ	Proteintech #24896-1-AP	1:1000
Anti CCTγ	Proteintech #10571-1-AP	1:1000
Anti CCTδ	Proteintech #21524-1-AP	1:1000
Anti CCTε	Proteintech #11603-1-AP	1:1000
Anti CCTζ	Proteintech #19793-1-AP	1:1000
Anti CCTη	Proteintech #15994-1-AP	1:1000
Anti CCTθ	Proteintech #12263-1-AP	1:1000
Goat anti-Rabbit (HRP)	Abcam #ab6721	1:2000
Anti-NPHS2	Proteintech#20384-1-AP20ul	1:1000(WB)/1:100(IF)
Goat anti-Rabbit AlexaFluor-647	Abcam#ab150083	1:1000
Anti-Flag AlexaFluor-488	BioLegend UK# 637317	1:1000 (IF)
Anti-Flag (HRP)	Sigma Aldrich#A8592	1:1000 (WB)
Anti-Myc tag [9E10]	Abcam#ab32	1:100 (IF)
Chicken anti-Mouse AlexaFluor-488	Abcam#ab150113	1:1000 (IF)

Supplementary Equation

Supplementary Equation 1. Baculovirus end-point dilution titration.

$$\frac{\mathrm{TU}}{\mathrm{ml}} = \left(Cn * \left(\frac{Tc}{100}\right)\right) * \frac{d}{Vv}$$

Cn = Cell number plated
Tc = Transduced cells percentage (e.g. eGFP+) – derived from flow-cytometry analysis
d = Viral dilutions (1-128)
Vv = Viral volume in ml

Supplementary Movie

Supplementary Movie S1. MultiMate-CellCycle BV transduced HeLa (provided as movie file)

Twelve-hours time-lapse confocal microscopy imaging of HeLa cells transduced with MultiMate-CellCycle BV. Images were acquired every 15 minutes. H2B-iRFP (top left, purple), mAG-hGem

(top right, green), mKO2-hCdt1 (bottom left, red) and Merge (bottom right) are displayed. Time stamp is in hours:minutes, Scalebar,50 μm.
