# Supplementary figures and images for: Highly efficient CRISPR-mediated large DNA docking and multiplexed prime editing using a single baculovirus
Source: Nucleic Acids Res. 2022 Jul 8;50(13):7783–99. doi: 10.1093/nar/gkac587 (PMC9303279; doi:10.1093/nar/gkac587)

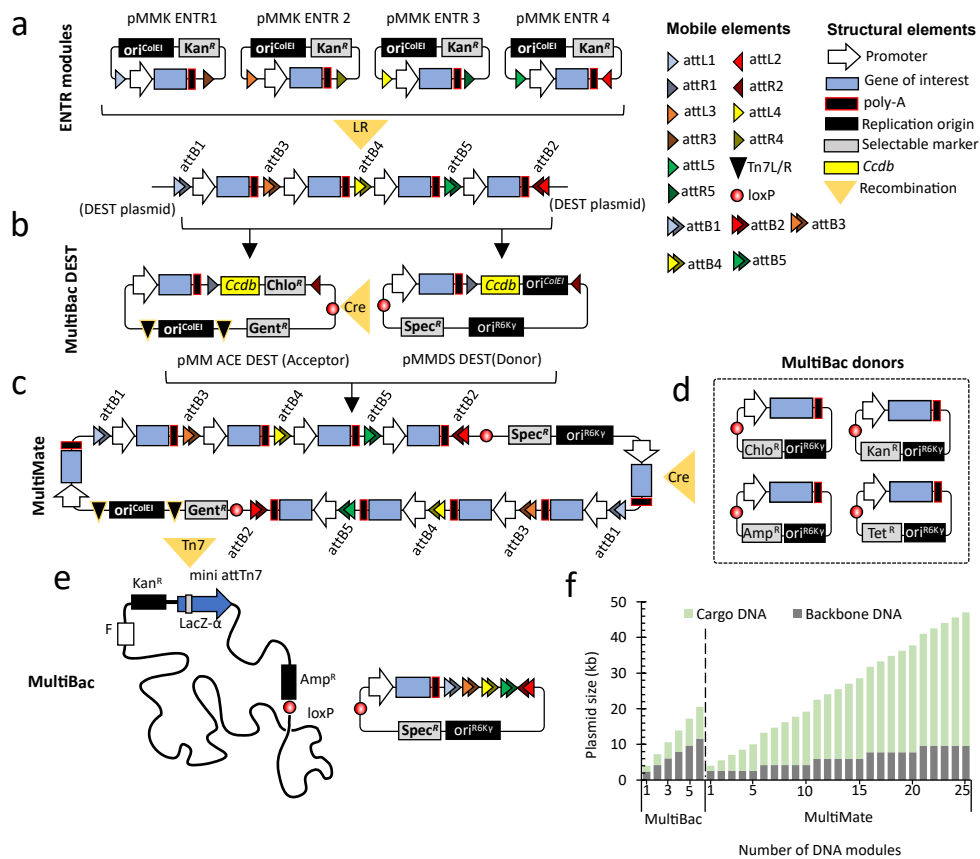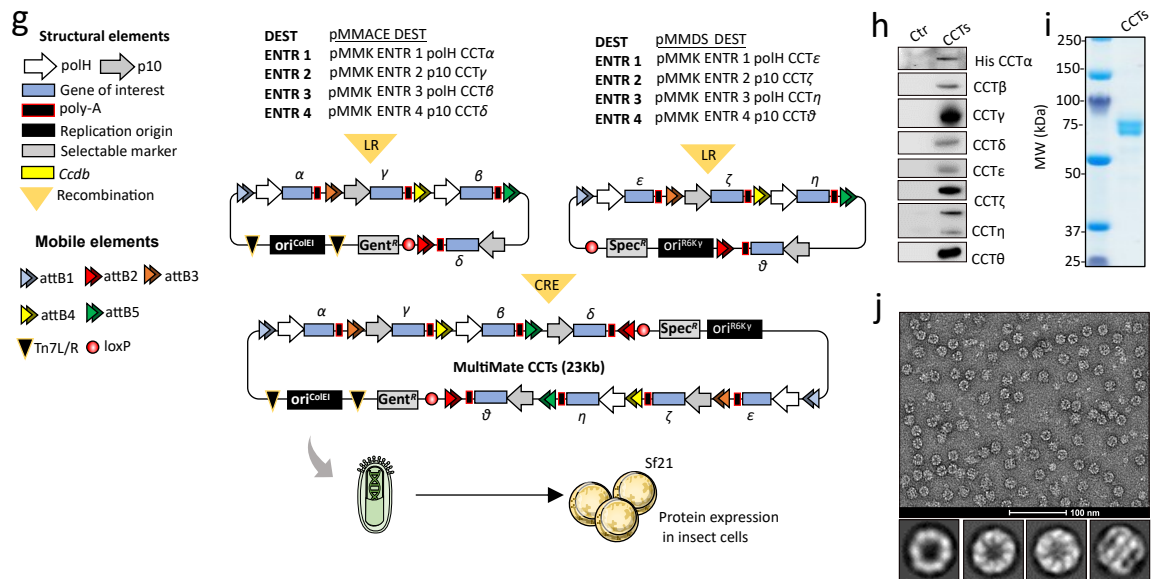

Supplement: gkac587_Supplemental_Files [file gkac587_supplemental_files.zip › Supplementary Figure S1.pdf]

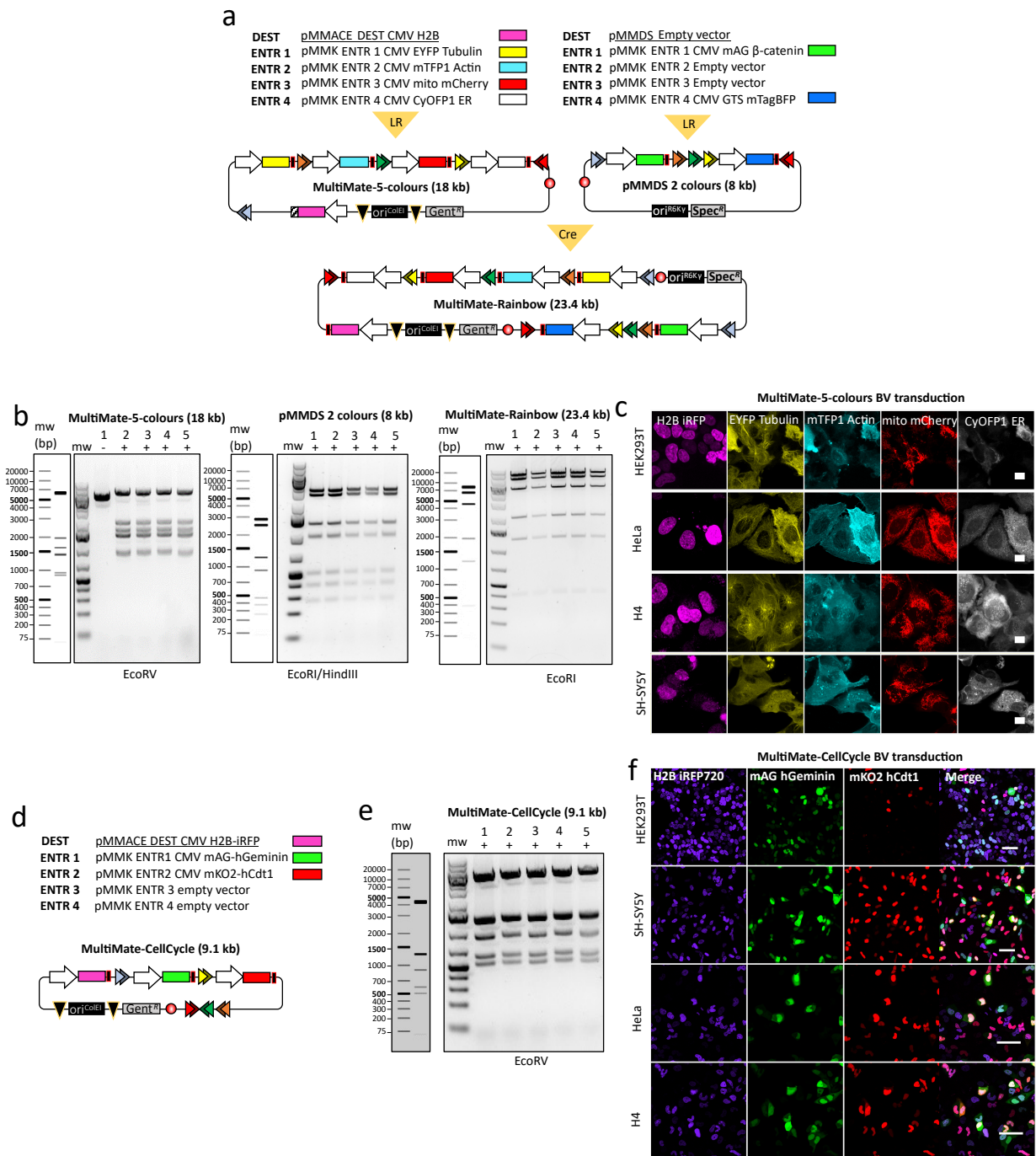

Supplement: gkac587_Supplemental_Files [file gkac587_supplemental_files.zip › Supplementary Figure S2.pdf]

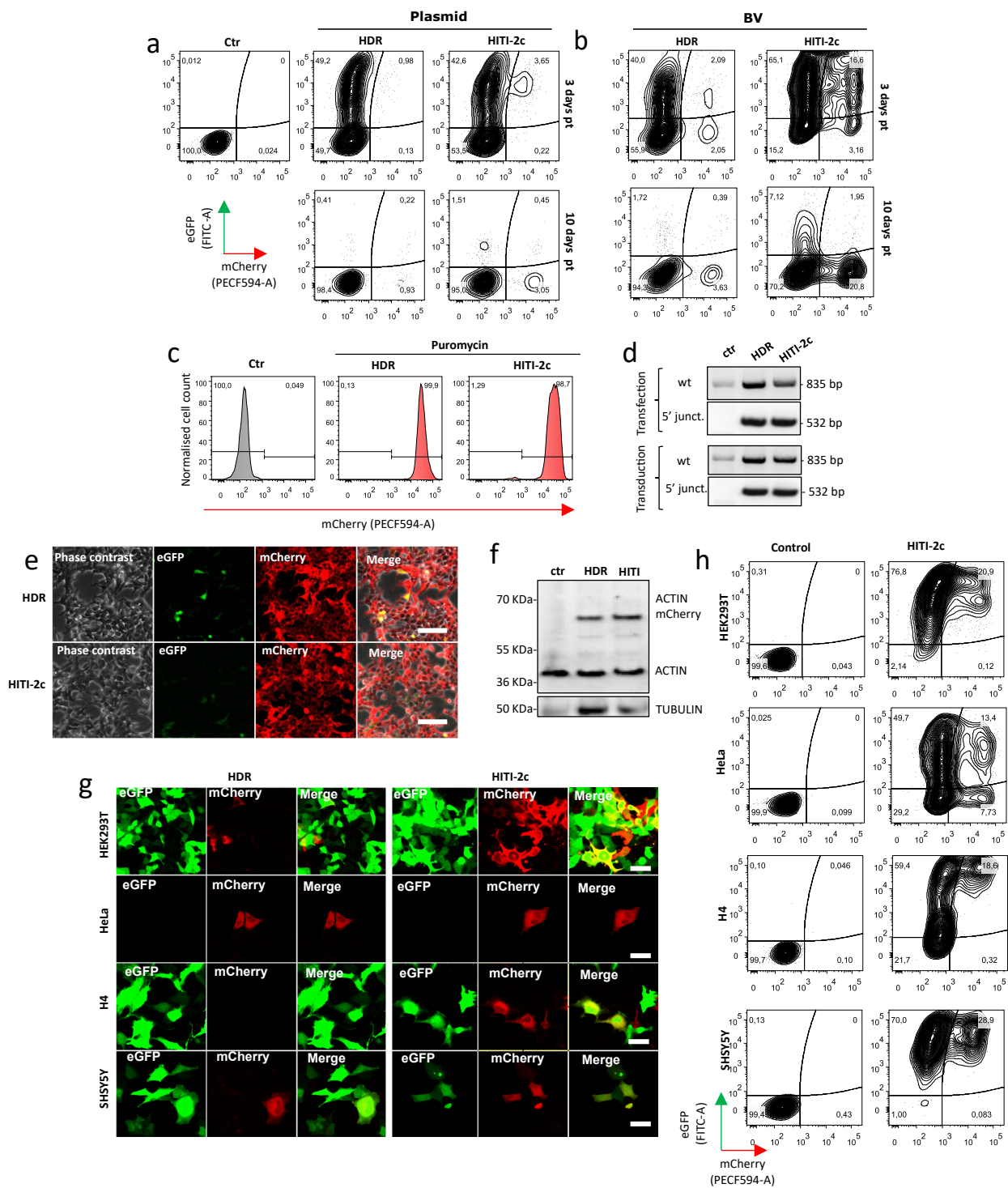

Supplement: gkac587_Supplemental_Files [file gkac587_supplemental_files.zip › Supplementary Figure S3.pdf]

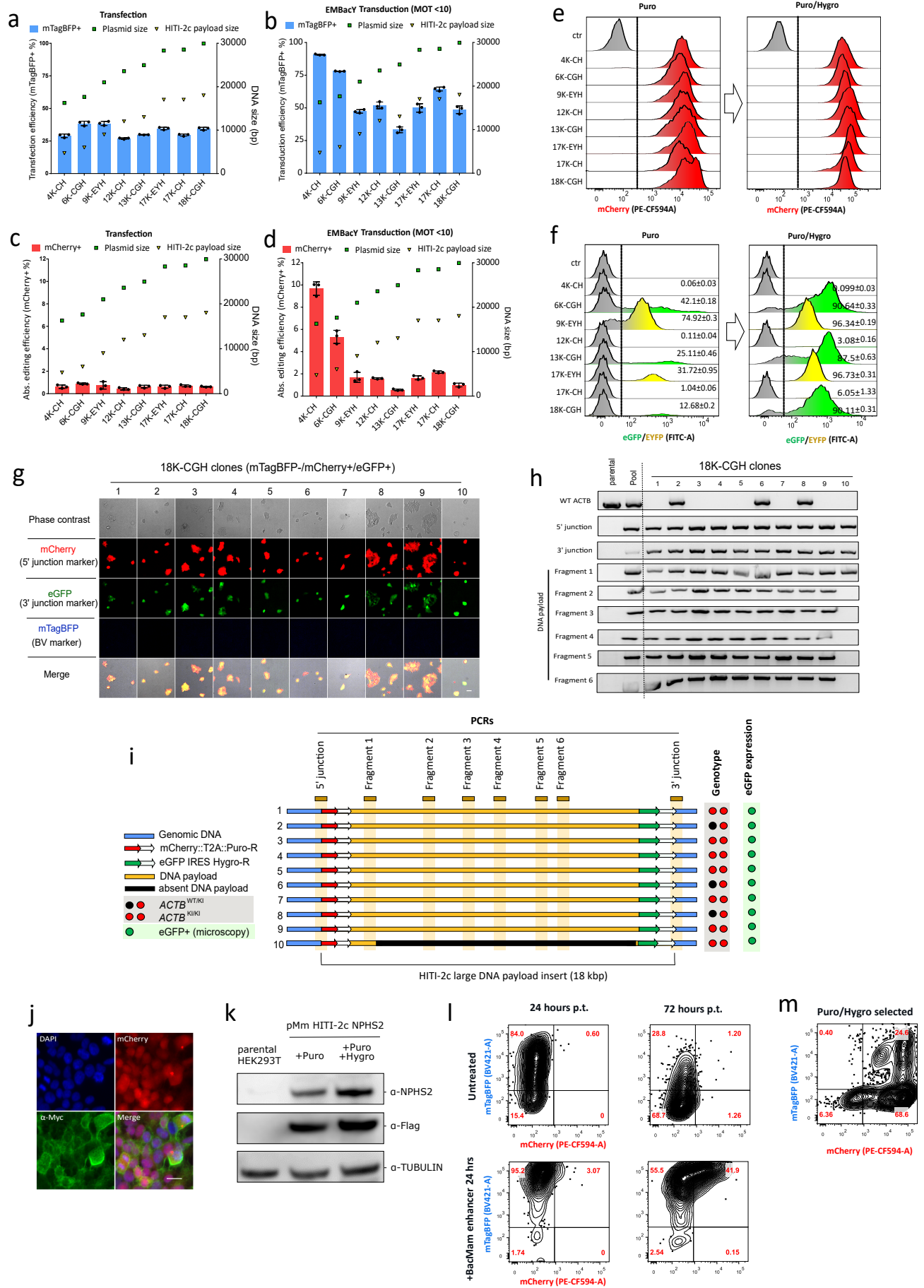

Supplement: gkac587_Supplemental_Files [file gkac587_supplemental_files.zip › Supplementary Figure S4.pdf]

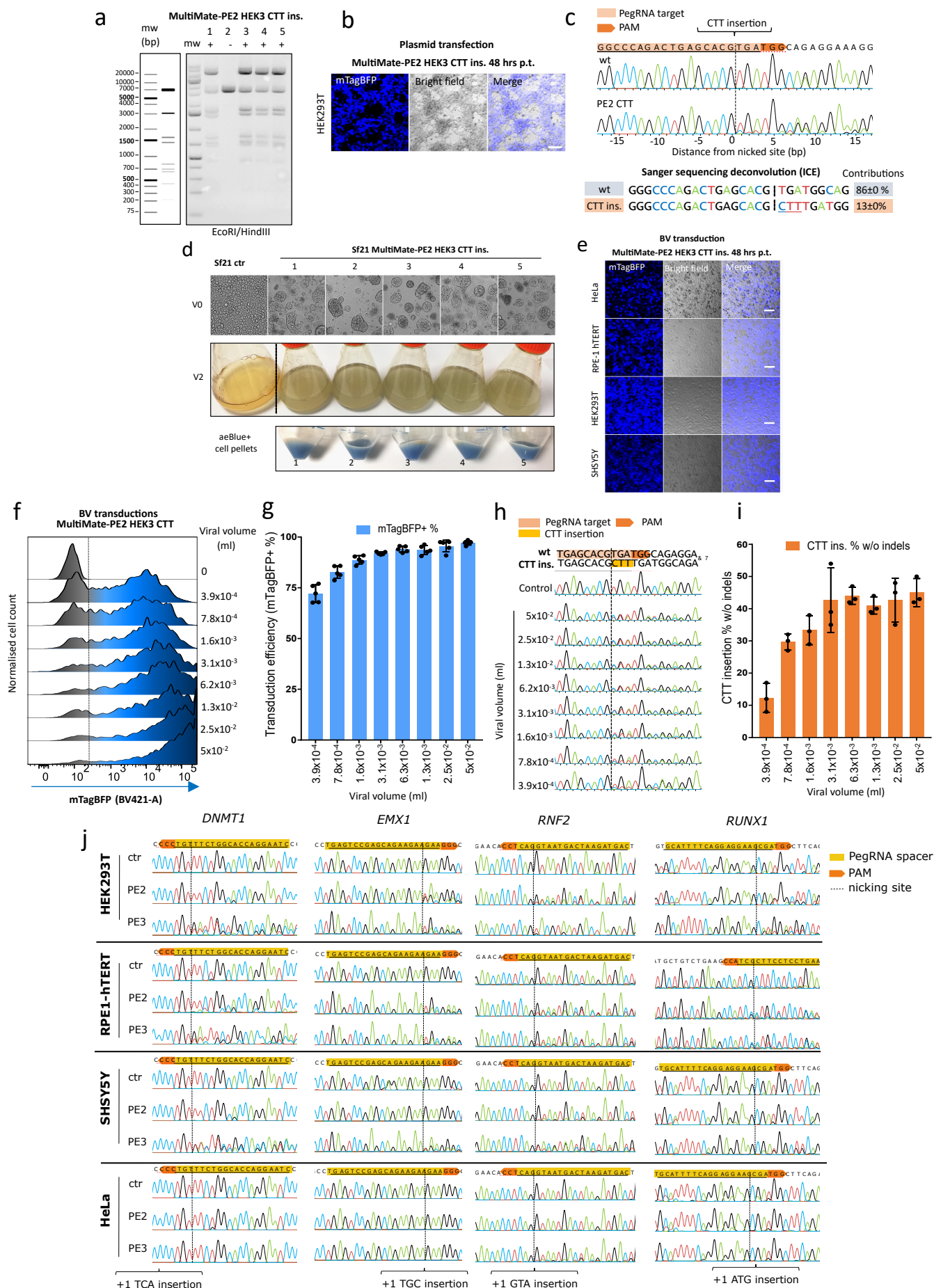

Supplement: gkac587_Supplemental_Files [file gkac587_supplemental_files.zip › Supplementary Figure S5.pdf]

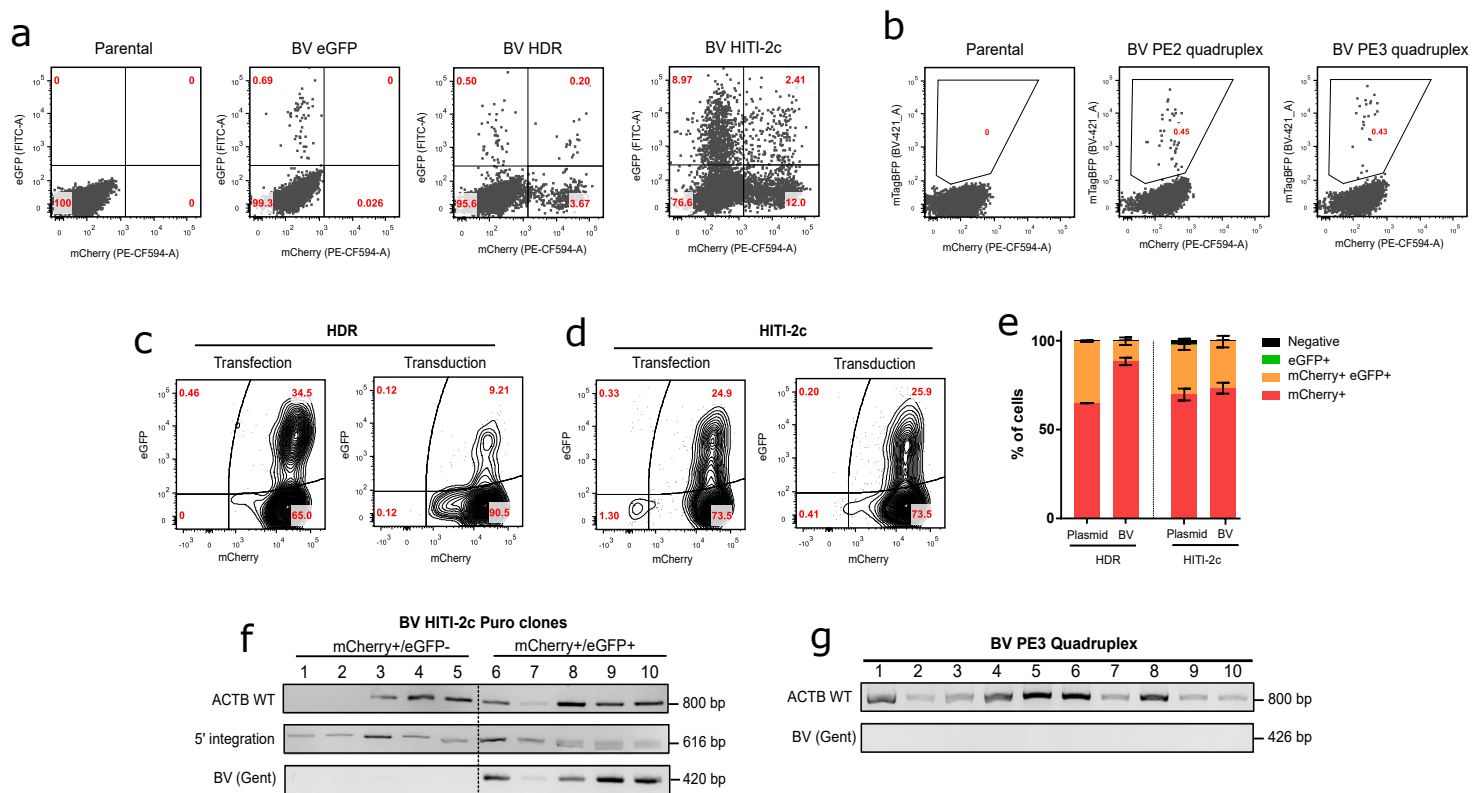

Supplement: gkac587_Supplemental_Files [file gkac587_supplemental_files.zip › Supplementary Figure S6.pdf]
